# Targeting the methyltransferase SETD8 impairs tumor cell survival and overcomes drug resistance independently of p53 status in multiple myeloma

**DOI:** 10.1186/s13148-021-01160-z

**Published:** 2021-09-16

**Authors:** Laurie Herviou, Sara Ovejero, Fanny Izard, Ouissem Karmous-Gadacha, Claire Gourzones, Celine Bellanger, Eva De Smedt, Anqi Ma, Laure Vincent, Guillaume Cartron, Jian Jin, Elke De Bruyne, Charlotte Grimaud, Eric Julien, Jérôme Moreaux

**Affiliations:** 1 IGH, CNRS, Univ Montpellier, Montpellier, France; 2 Laboratory for Monitoring Innovative Therapies, Department of Biological Hematology, CHU Montpellier, Montpellier, France; 3 Institut de Recherche en Cancérologie de Montpellier (IRCM), INSERM U1194, Institut Régional du Cancer (ICM), 34298 Montpellier, France; 4 University of Montpellier, 34090 Montpellier, France; 5 Department of Hematology and Immunology-Myeloma Center Brussels, Vrije Universiteit Brussel, Brussels, Belgium; 6 Mount Sinai Center for Therapeutics Discovery, Departments of Pharmacological Sciences and Oncological Sciences, Tisch Cancer Institute, Icahn School of Medicine at Mount Sinai, New York, NY 10029 USA; 7 Department of Clinical Hematology, CHU Montpellier, Montpellier, France; 8 Centre National de La Recherche Scientifique (CNRS), 34293 Montpellier, France; 9 Institut Universitaire de France (IUF), Paris, France

**Keywords:** multiple myeloma, SETD8, SET8, Histone methylation, p53

## Abstract

**Background:**

Multiple myeloma (MM) is a malignancy of plasma cells that largely remains incurable. The search for new therapeutic targets is therefore essential. In addition to a wide panel of genetic mutations, epigenetic alterations also appear as important players in the development of this cancer, thereby offering the possibility to reveal novel approaches and targets for effective therapeutic intervention.

**Results:**

Here, we show that a higher expression of the lysine methyltransferase SETD8, which is responsible for the mono-methylation of histone H4 at lysine 20, is an adverse prognosis factor associated with a poor outcome in two cohorts of newly diagnosed patients. Primary malignant plasma cells are particularly addicted to the activity of this epigenetic enzyme. Indeed, the inhibition of SETD8 by the chemical compound UNC-0379 and the subsequent decrease in histone H4 methylation at lysine 20 are highly toxic in MM cells compared to normal cells from the bone marrow microenvironment. At the molecular level, RNA sequencing and functional studies revealed that SETD8 inhibition induces a mature non-proliferating plasma cell signature and, as observed in other cancers, triggers an activation of the tumor suppressor p53, which together cause an impairment of myeloma cell proliferation and survival. However, a deadly level of replicative stress was also observed in p53-deficient myeloma cells treated with UNC-0379, indicating that the cytotoxicity associated with SETD8 inhibition is not necessarily dependent on p53 activation. Consistent with this, UNC-0379 triggers a p53-independent nucleolar stress characterized by nucleolin delocalization and reduction of nucleolar RNA synthesis. Finally, we showed that SETD8 inhibition is strongly synergistic with melphalan and may overcome resistance to this alkylating agent widely used in MM treatment.

**Conclusions:**

Altogether, our data indicate that the up-regulation of the epigenetic enzyme SETD8 is associated with a poor outcome and the deregulation of major signaling pathways in MM. Moreover, we provide evidences that myeloma cells are dependent on SETD8 activity and its pharmacological inhibition synergizes with melphalan, which could be beneficial to improve MM treatment in high-risk patients whatever their status for p53.

**Supplementary Information:**

The online version contains supplementary material available at 10.1186/s13148-021-01160-z.

## Introduction

Multiple Myeloma (MM) is a cancer of terminally differentiated plasma cells [1] and is the second most common hematological malignancy [2]. Over the past 15 years, several advances in treatments have led to a significantly higher survival of intensively treated patients [3]. Unfortunately, despite the recent progress in MM treatments, most patients will ultimately relapse and develop drug resistance.

In addition to a wide panel of genetic mutations, recent studies have pinpointed that epigenetic alterations, including aberrant DNA and histone methylation, might be also important players in MM development [4]. These recent findings could illuminate new mechanisms central to the genesis of MM and offer the possibility to reveal novel approaches and targets for effective therapeutic intervention. For example, the inhibition of the histone H3K27 methyltransferase EZH2 has recently emerged as a potential strategy for the treatment of myeloma [5]. Moreover, classic epigenetic modulating agents, such as histone deacetylase and DNA methyltransferase inhibitors, are already tested as monotherapy or in combination with conventional anti-MM agents [6, 7]. However, the occurrence of important side effects and the appearance of resistance to these drugs increase the need to identify novel epigenetic targets in MM and evaluate their pre-clinical perspective.

SETD8 (also known as SET8, PR-Set7, KMT5A) has been identified as the epigenetic enzyme responsible for the mono-methylation of histone H4 at lysine 20 (H4K20me1) [8]. SETD8 and H4K20me1 are naturally increased during mitosis and play a critical role in chromatin compaction, gene regulation and cell-cycle progression [8–10]. In addition, SETD8 could induce the methylation of non-histone proteins, such as the replication factor PCNA and the tumor suppressor p53 [11, 12]. While SETD8-mediated methylation of p53 inhibits apoptosis [12], PCNA methylation by SETD8 might enhance the interaction with the Flap endonuclease FEN1 and promote cell proliferation [11]. Consistent with this, the overexpression of SETD8 has been reported in many different solid tumors [14–17] and pharmacological inhibition of SETD8 is sufficient to activate the p53 pro-apoptotic program in neuroblastoma cell lines [18]. This has suggested that this enzyme could be an attractive target to rescue p53 functions in cancers displaying a low incidence of *TP53* genetic alterations, as it is the case at early stages in MM [19]. However, the role of SETD8 and its incidence in the development of MM or any hematological malignancies is not known.

Here, we provide evidence that malignant plasma cells are addicted to SETD8 expression, which is associated with a poor outcome independently of changes in the steady state level of histone H4K20me1. Although inducing p53 canonical pathway, we show that the pharmacological inhibition of SETD8 by the chemical compound UNC-0379 causes specific nucleolar alterations and cytotoxic effects independently of p53 status. Finally, the combination of UNC-0379 with the cytotoxic agent melphalan strongly enhances DNA damage and overcomes drug resistance, suggesting that targeting SETD8 activity could be beneficial to improve MM treatment in high-risk patients independently of their mutational status for p53.

## Materials and methods

### Primary multiple myeloma cells

Patients’ MMCs were purified using anti-CD138 MACS microbeads (Miltenyi Biotec, Bergisch Gladbach, Germany) and their gene expression profile (GEP) obtained using Affymetrix U133 plus 2.0 microarrays as described [20–22]. We also used publicly available Affymetrix GEP (Gene Expression Omnibus, accession number GSE2658) of a cohort of 345 purified MMC from previously untreated patients from the University of Arkansas for Medical Sciences (UAMS, Little Rock, AR), termed in the following UAMS-TT2 cohort. These patients were treated with total therapy 2 including high-dose melphalan (HDM) and autologous stem cell transplantation (ASCT) [23]. We also used Affymetrix data from total therapy 3 cohort (UAMS-TT3; *n* = 158; E-TABM-1138) [24]. Mouse 5T33MMvv cells originated spontaneously in aging C57BL/KaLwRij mice and have since been propagated in vivo by intravenous transfer of diseased marrow in young syngeneic mice as described [25, 26].

### Treatment of primary MM cells

Bone marrow of patients with previously untreated MM (*n* = 8) at the university hospital of Montpellier was obtained after patients’ written informed consent in accordance with the Declaration of Helsinki and agreement of the Montpellier University Hospital Centre for Biological Resources (DC-2008-417). Mononuclear cells were treated with or without UNC-0379 (1 μM, 2.5 μM or 5 μM) and MMC cytotoxicity was evaluated using anti-CD138-phycoerythrin monoclonal antibody (Immunotech, Marseille, France) as described [27].

### Apoptosis assays

Cells were cultured in 24-well, flat-bottomed microtiter plates at 10^5^ cells per well in RPMI1640–10% FCS or X-VIVO 20 culture media with or without IL-6 (3 ng/mL) and appropriate concentration of chemical drugs. After 4 days of culture, cells were washed twice in PBS and apoptosis was assayed with PE-conjugated Annexin V labeling (BD Biosciences) using a Fortessa flow cytometer (BD) following manufacturer's protocols.

### Human myeloma cell lines (HMCLs)

XG human myeloma cell lines (HMCLs) were cultured in the presence of recombinant IL-6 as previously described [28]. XG7 Melphalan-resistant cell line was derived from the sensitive parental XG7 cells [29]. JJN3 was kindly provided by Dr Van Riet (Brussels, Belgium), JIM3 by Dr MacLennan (Birmingham, UK) and MM1S by Dr S. Rosen (Chicago, USA). AMO-1, LP1, L363, U266, OPM2, and SKMM2 were purchased from DSMZ (Braunsweig, Germany) and RPMI8226 from ATTC (Rockville, MD, USA). HMCLs were authenticated according to their short tandem repeat profiling and their gene expression profiling using Affymetrix U133 plus 2.0 microarrays deposited in the ArrayExpress public database under accession numbers E-TABM-937 and E-TABM-1088 [28].

### Establishment of shRNA expressing HMCLs

Control and p53 shRNA sequences were cloned in the pLenti4-EZ-mIR plasmid as previously described [30]. SETD8 and associated control shRNA sequences were cloned into a puromycin retroviral vector RNAi Ready pSiren as described [31]. Retroviral particles were produced in 293FT cells. Briefly, 293FT cell line was cultured in Dulbecco's modified Eagle's medium and supplemented with 10% defined fetal bovine serum, 500 µg/mL geneticin, 4 mM *L*-glutamine, and 1 mM MEM sodium pyruvate. The day before transfection, cells were plated into a 10 cm tissue culture plate to 90%-95% confluence. 9 µg of ViraPower packaging mix (Invitrogen) and 9 µg of lentiviral plasmids were co-transfected into 293FT cells using 36 µL Lipofectamine 2000 reagent (Invitrogen). Forty-eight hours later, culture supernatants were collected, concentrated 100-fold by ultracentrifugation (20,000 g; 4 h) and viral titers determined. Corresponding HMCLs were transduced with virus and stable transduced cells were obtained by adding zeocin (10 µg/mL) for pLenti4-EZ-mIR and puromycin (2.5 µg/mL) for pSIREN viral particles.

### Growth assays and cell cycle analysis

Cells were cultured for 4 days in 96-well flat-bottom microtiter plates in RPMI 1640 medium, 10% FCS, and 2 ng/ml IL-6 (control medium) in the presence of UNC-0379. Cell growth was evaluated by quantifying intracellular ATP amount with a Cell Titer Glo Luminescent Assay (Promega, Madison, WI) using a Centro LB 960 luminometer (Berthold Technologies, Bad Wildbad, Germany). For cell cycle analysis, cells were cultured in 24-well flat-bottomed microtiter plates at 10^5^ cells per well in RPMI1640–10% FCS or X-VIVO 20 culture media with or without IL-6 (3 ng/mL). The cell cycle was assessed using DAPI staining (Sigma-Aldrich, Saint-Louis, MO, USA) and cells in the S phase using incubation with bromodeoxyuridine (BrdU) for 1 h and labeling with an anti-BrdU antibody (APC BrdU flow kit, BD Biosciences, San Jose, CA, USA) according to the manufacturer’s instructions. Flow cytometry analysis was done on a Fortessa flow cytometer (BD, Mountain View, CA, USA).

### RNA sequencing

HMCLs were cultured for 16 h with or without 5 µM of UNC-0379, or infected with shControl or shRNA SETD8 pSIREN vectors for 48 h. RNA samples were collected as previously described. The RNA sequencing (RNA-seq) library preparation was done with 150 ng of input RNA using the Illumina TruSeq Stranded mRNA Library Prep Kit. Paired-end RNA-seq were performed with Illumina NextSeq sequencing instrument (Helixio, Clermont-Ferrand, France). RNA-seq read pairs were mapped to the reference human GRCh37 genome using the STAR aligner [32]. All statistical analyses were performed with the statistics software R (version 3.2.3; available from https://www.r-project.org) and R packages developed by BioConductor project (available from https://www.bioconductor.org/) [33]. The expression level of each gene was summarized and normalized using DESeq2 R/Bioconductor package [34]. Differential expression analysis was performed using DESeq2 pipeline. *p* values were adjusted to control the global FDR across all comparisons with the default option of the DESeq2 package. Genes were considered differentially expressed if they had an adjusted *p* value of 0.05 and a fold change of 1.5. Pathway enrichment analyses were performed using online the curated gene set collection on the Gene Set Enrichment Analysis software (http://software.broadinstitute.org/gsea/msigdb/index.jsp) [34, 35].

### Gene expression profiling and statistical analyses

Gene expression data were normalized with the MAS5 algorithm and analyses processed with GenomicScape (http://www.genomicscape.com) [37] the R.2.10.1 and bioconductor version 2.5 programs [33]. Gene Set Expression Analysis (GSEA) was used to identify genes and pathways differentially expressed between populations. Univariate and multivariate analysis of genes prognostic for patients’ survival was performed using the Cox proportional hazard model. Difference in overall survival between groups of patients was assayed with a log-rank test and survival curves plotted using the Kaplan–Meier method (Maxstat R package) [38].

### 53BP1 staining-immunofluorescence microscopy

After deposition on slides using a Cytospin centrifuge (600 r.p.m. for 10 min), cells were fixed with 4% PFA, permeabilized with 0.5% Triton in PBS and saturated with 5% bovine milk in PBS. The rabbit anti-53BP1 (Novus Biologicals—Littleton, CO, USA—NB100304) antibody was diluted 1/300 in 5% bovine milk in PBS, and deposited on cytospins for 60 min at room temperature. Slides were washed twice and anti-rabbit alexa 488-conjugated antibody (diluted 1/500 in 5% bovine milk in PBS) was added for 60 min at room temperature. Slides were washed and mounted with Vectashield and 1% DAPI. Images and fluorescence were captured with a ZEISS Axio Imager Z2 microscope (X63 objective) (Oberkochen, Germany), analyzed with Omero (omero.mri.cnrs.fr) server and ImageJ software.

### Nucleolar proteins staining-immunofluorescence microscopy

After indicated treatments and deposition on slides as described above, cells were fixed with 4% PFA for 10 min at RT, permeabilized with 0.1% Triton in PBS-0.5% BSA for 10 min, and saturated with PBS-1% BSA for 20 min. Anti-nucleolin (ab22758, Abcam) and anti-fibrillarin (ab5821, Abcam) antibodies were diluted 1/200 in saturation buffer, and incubated for 90 min in a humid chamber at room temperature. Slides were washed 3 times with PBS-0.01% Tween-20 and anti-rabbit Alexa Texas Red-conjugated antibody (diluted 1/500 in saturation buffer) was added for 45 min in a humid chamber at room temperature protected from light. Slides were washed 3 times (5 min each) as previously, incubated with DAPI (20 µg/mL in water) for 5 min protected from light, washed 3 times with water, air dried and mounted with ProLong (Invitrogen). Images and fluorescence were captured with a ZEISS Axio Imager Z1 microscope (X40 objective) (Oberkochen, Germany), and analyzed with Omero (omero.mri.cnrs.fr) server. Quantification of cells with nucleolin delocalization was visually performed.

### 5-Ethynyl uridine (EU) staining-immunofluorescence mycroscopy

Cells were treated with UNC-0379 3 µM or the vehicle DMSO (0.0003%) for 24 h. EU (0.2 mM) was added during the last 5 min of treatment. Cells were deposited on slides as described above, fixed with 4% PFA for 10 min at RT, permeabilized with 0.5% Triton in PBS-0.5% BSA for 10 min, saturated with PBS-1% BSA for 20 min and Click-it reaction was performed. Briefly, for 10 slides, a mix of 438 µL PBS 1X + 15 µL CuSO_4_ (0.1 M) + 2.5 µL Azide AF555 + 50 µL Vitamine C (100 mM) was prepared by adding the reagents in the mentioned order. Slides were incubated in a humid chamber protected from light for 15 min at RT, washed 3 times with PBS-0.01% Tween-20, and subsequently incubated with DAPI and mounted as described above. Image acquisition was performed with a ZEISS Axio Imager Z1 microscope (X40 objective) (Oberkochen, Germany), and analyzed with Omero (omero.mri.cnrs.fr) server. Quantification of EU intensity was performed with CellProfiler™ “Human Cell” pipeline.

#### Immunoblot analysis

Cells washed with phosphate-buffered saline (PBS) were lysed in SDS buffer and boiled at 94 °C for 5 min. After measuring protein quantity by Bradford, equal amounts of protein were resolved by SDS-PAGE, transferred to a nitrocellulose membrane (Millipore) and probed with one of the following antibodies: mouse anti-Chk1 (1:1000, abcam), rabbit anti-p21 (1:500, Cell signaling), rabbit anti- p53 (1:1000, Cell Signaling), rabbit anti-SETD8 (1:1000, Cell Signaling), mouse anti-β-actin (1:20,000, Sigma), rabbit anti-H2A.X and anti-phospho-H2A.X-Ser139 (1:1000, Cell signaling), rabbit anti-H4-K20me1 (1:1000 Cell Signaling), and rabbit anti-Histone H4 (1:1000, Cell Signaling), rabbit anti-tubulin (1:1000, Cell signaling). Membranes were then incubated with the appropriate horseradish peroxidase (HRP)-conjugated secondary antibodies. The immunoreactive bands were detected by chemiluminescence (Pierce).

### Drug combination study

The interaction between the drugs tested in vitro was investigated with a concentration matrix test, in which increasing concentration of each single drug were assessed with all possible combinations of the other drugs. For each combination, the percentage of expected growing cells in the case of effect independence was calculated according to the Bliss equation (Combes et al., 2019; Greco et al., 1995):

$$fuC = fuA \cdot fuB$$

where fuC is the expected fraction of cells unaffected by the drug combination in the case of effect independence, and fuA and fuB are the fractions of cells unaffected by treatment A and B, respectively. The difference between the fraction of living cells in the cytotoxicity test and the fuC value was considered as an estimation of the interaction effect, with positive values indicating synergism and negative values antagonism.

Synergy matrix was built with the R package “SynergyFinder”.

### In vitro plasma cell differentiation

Peripheral blood cells from healthy donors were purchased from the French Blood Center (Toulouse, France) and CD19 + CD27 + MBCs purified (> 95% purity) using “MBC isolation kit” (Order no. 130-093-546, Miltenyi) according to manufacturer’s instructions. Preplasmablasts (prePB), plasmablasts (PB) and plasma cells (PC) were generated as reported [39, 40], by a 3-step in vitro protocol. At day 7 of differentiation, when PB was the most abundant population in the culture, UNC-0379 (at 1, 2.5 or 5 µM) or the vehicle DMSO (= mock, at 0.0005%) were added, and cells were incubated for the next 3 days during their differentiation into PC. At the end of this treatment, all cells were collected, visually counted with Trypan Blue to asses viability, apoptosis was analyzed as described above, and PCs (CD38 + CD138 + cells) were quantified by cytometry. Data are expressed as percentage of CD38 + CD138 + cells compared to the mock sample (100%).

## Results

### SETD8 up-regulation in myeloma is associated with a poor outcome

**Fig. 1:**
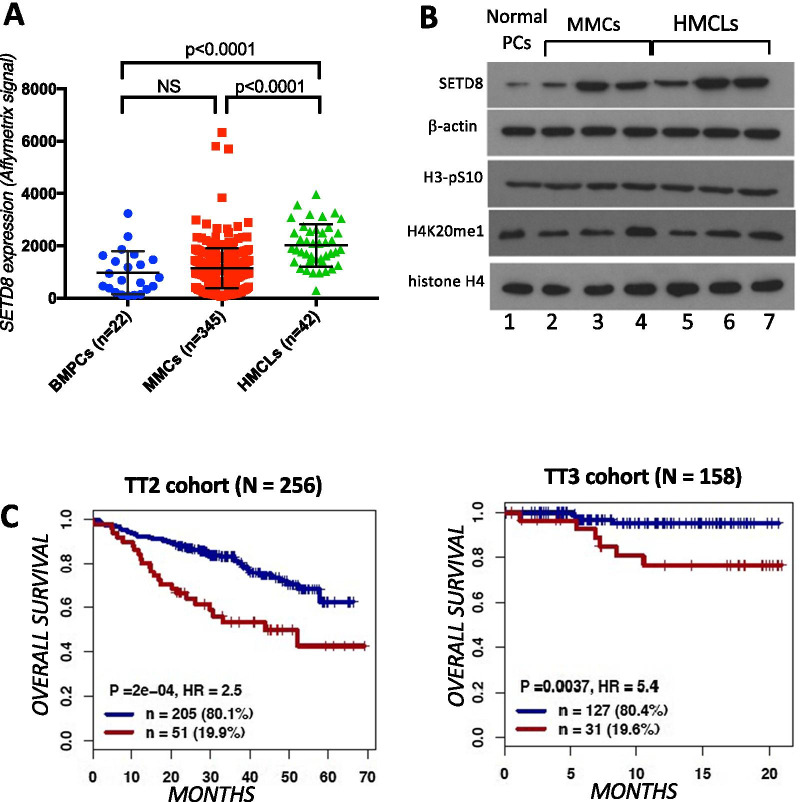
*SETD8* expression is a prognosis value in MM. **a** *SETD8* gene expression in BMPCs, patients’ MMCs and HMCLs. Data are MAS5-normalized Affymetrix signals (U133 plus 2.0 microarrays). Statistical difference was assayed using a student *t* test. **b** Immunoblot analysis of indicated proteins in total lysates from normal plasma cells, MMCs and HMCLs (XG1, XG7, XG25). β-actin and histone H4 were used as loading controls. **c** Overall survival and of newly diagnosed MM patients (UAMS-TT2, *N* = 345; and UAMS-TT3, *N* = 158) who’s MMCs highly expressed *SETD8* gene. The splitting of the patients into two groups according to *SETD8* expression in MMCs was done using the Maxstat algorithm

In order to identify epigenetic factors potentially involved in MM, we used public Affymetrix microarrays gene expression data sets to identify which genes encoding histone-modifying enzymes are differentially expressed between normal bone marrow plasma cells (BMPCs, *n* = 22), purified primary MM cells from newly diagnosed patients (MMCs, *n* = 345) and human myeloma cell lines (HMCLs, *N* = 42)^19^. As shown in Fig. 1a, significantly higher levels of mRNAs encoding the histone H4K20 mono-methyltransferase SETD8 were found in HMCLs compared to BMPCs and MMCs (Fig. 1a). Furthermore, although we did not observe a statistical difference with BMPCs, *SETD8* mRNA levels appeared heterogeneous in MMCs (Fig. 1a) ranging from 65 to 63,338 in Affymetrix signal. These contrasted *SETD8* mRNA levels were not restrained to a particular MM molecular sub-type (Additional files 2 and 3: Figure S1A). Immunoblot analysis with a specific SETD8 antibody showed that HMCLs and some MMCs displayed higher SETD8 protein levels compared with normal plasma cells (PC) (Fig. 1b). These higher SETD8 protein levels were independent of the natural cell-cycle fluctuation of the enzyme [9], as the levels of the mitotic marker histone H3-S10 phosphorylation were relatively similar in all tested samples (Fig. 1b). SETD8 is known as the unique enzyme responsible for the mono-methylation of histone H4 at lysine 20 (H4K20me1) [8]. Yet, levels of H4K20me1 remained roughly unchanged in HMCLs and MMCs displaying higher SETD8 levels and similar levels of the G2/M marker histone H3-S10 phosphorylation (Fig. 1b), indicating that SETD8 up-regulation is not accompanied by a significant increase in the steady state level of histone H4K20me1 in MM.

We next investigated the prognostic value of *SETD8* up-regulation in two independent cohorts of previously untreated MM patients (UAMS-TT2 *n* = 256 and UAMS-TT3 *n* = 158 cohorts), using Maxstat R algorithm. High *SETD8* mRNA levels were predictive of both shorter event free survival and overall survival in the two cohorts (Fig. 1c). Consistent with these results, *SETD8* mRNA levels were significantly increased in patients harboring Chr1q21 gain (Additional files 2 and 3: Figure S1B) or presenting a high gene expression-based proliferation index (GPI) (Additional files 2 and 3: Figure S1C)*,* molecular features being associated with a poor outcome in MM patients*.* However, *SETD8* expression did not correlate with MMC plasma cell labeling index (PCLI) in a cohort of 101 newly diagnosed patients (Additional files 2 and 3: Figure S1D), suggesting that *SETD8* up-regulation is likely not a hallmark of MMC proliferation status. Gene set expression analysis (GSEA) of patients with high *SETD8* expression highlighted a significant enrichment of genes involved in IRF4 targets, MYC-MAX targets, MAPK pathway and DNA repair (Additional files 2 and 3: Figure S2, *p* < 0.001), suggesting that *SETD8* up-regulation correlates with changes in signaling pathways involved in MM pathophysiology. Furthermore, *SETD8* expression is significantly higher in MM cells from relapsed patients compared to newly diagnosed patients, underlining a potential role of SETD8 in drug resistance (Additional files 2 and 3: Figure S3). Altogether, these data reveal that the epigenetic enzyme SETD8 is overexpressed in myeloma and this up-regulation is associated with a poor outcome and deregulation of major signaling pathways in MM patients.

### UNC-0379-mediated SETD8 inhibition leads to cell-cycle defects and apoptosis in MMCs

**Fig. 2:**
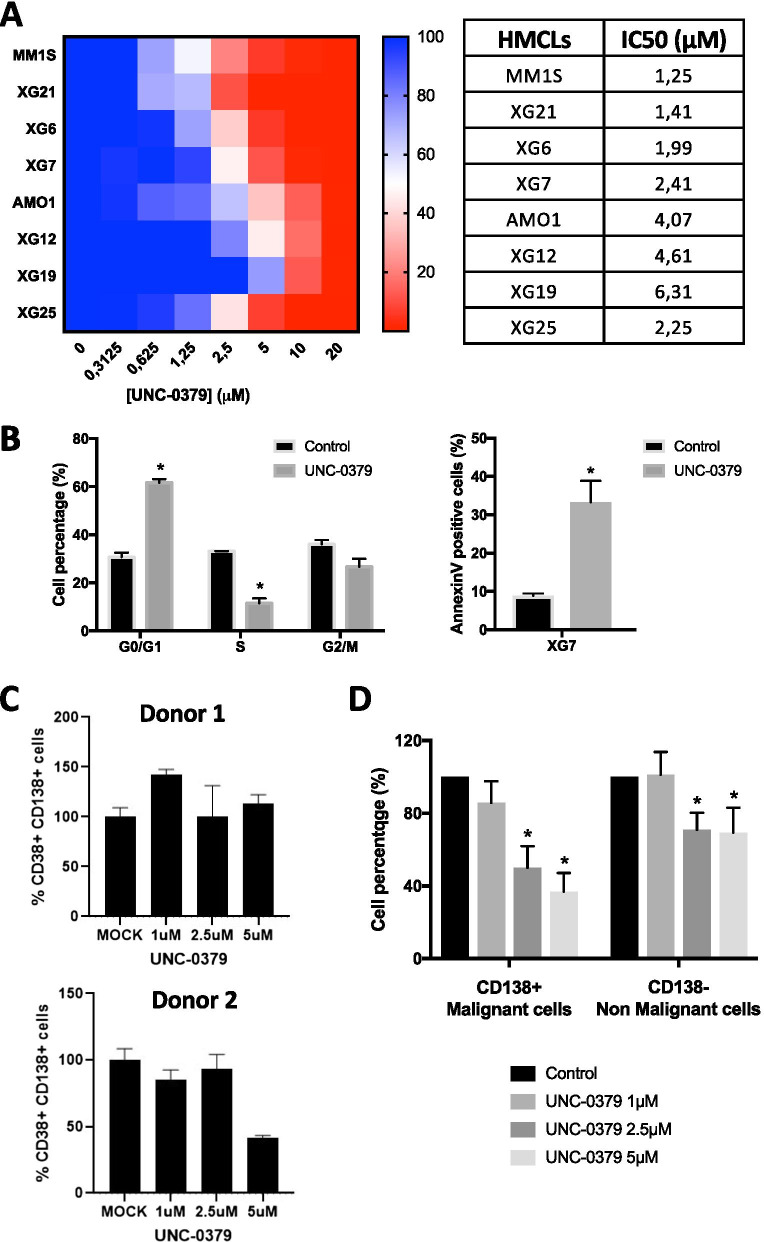
SETD8 inhibitor UNC-0379 is highly toxic to malignant plasma cells. **a** Graphical representation of HMCLs viability upon exposure to various concentrations of UNC-0379. Color scale represents cell viability, from the highest (blue) to the lowest (red) values. The IC50 value of each tested HMCL is indicated. Data are mean values ± standard deviation (SD) of five experiments determined on sextuplet culture wells. **b** The first bar graph results from the quantitation of cell cycle distribution of control (untreated) and UNC-0379-treated XG7 and XG25 HMCLs 48 h after treatment. After short-pulse of BrdU incorporation, cell cycle was analyzed by FACS using DAPI and anti-BrdU antibody. Second bar graph represents the results from the quantitation of apoptosis in control and UNC-0379-treated XG7 and XG25 HMCLs by flow cytometry with AnnexinV-PE staining at 96 h after UNC-0379 treatment. Data shown are mean values ± SD of 4 separate experiments. Statistical analysis was done with a paired *t* test. (*) indicates a significant difference compared to control cells using a Wilcoxon test for pairs (*p* ≤ 0.05). **c** Percentage of normal mature plasma cells (CD38 + CD138 +) from two different donors and quantified by FACS after in vitro treatment with increasing concentrations of UNC-0379 or the vehicle DMSO at the highest concentration (MOCK) for 4 days. Samples were treated and analyzed in duplicates. **d** Percentage of in vitro cultivated primary MM cells (CD138 + tumor cells) and bone marrow microenvironment (CD138- non-malignant cells) upon increasing concentrations of UNC-0379 treatment for 4 days. Data shown are mean values of 8 patient samples. *Indicates a significant difference compared to control cells using a Wilcoxon test for pairs (*p* ≤ 0.05)

To determine the biological significance of *SETD8* up-regulation in MM pathophysiology, the effects of the small-molecule SETD8 inhibitor UNC-0379 were examined in eight different HMCLs representative of the disease. UNC-0379 is a well-characterized substrate-competitive inhibitor selective for SETD8 [15, 18, 42]. As shown in Fig. 2a, UNC-0379 treatment was sufficient to inhibit the growth of all HMCLs in a dose dependent manner with an average half maximal inhibitory concentration (IC_50_) ranging from 1.25 to 6.3 µM (Fig. 2a). To determine the molecular mechanisms of this HMCL growth inhibition, the effects of UNC-0379 treatment on SETD8 activity, cell proliferation and survival were examined using immunoblot and flow cytometry assays in XG7 and XG25 MM cell lines, which displayed similar IC_50_ values for UNC-0379. Analysis of whole cell extracts after 24 h of treatment showed a strong decrease in H4K20me1, thereby demonstrating the rapid and efficient inhibition of SETD8 activity (Additional files 2 and 3: Figure S4A). In the following hours, this SETD8 inhibition was associated with cell-cycle defects, as shown in both cell lines by an accumulation in G1 phase and a decrease in DNA replication (S) phase 48 h after treatment (Fig. 2b and Additional files 2 and 3: Figure S4B). At later time points, these cell cycle defects were followed by the activation of apoptosis, as measured by the appearance of 33% and 26% of annexin-V positive cells in XG7 and XG25 cell lines respectively (Fig. 2b and Additional files 2 and 3: Figure S4C).

To demonstrate that SETD8 is indeed required for growth and viability of malignant plasma cells, bone marrow (BM) cells isolated from two healthy donors and from eight MM patients were cultured with recombinant interleukin 6 in presence or not of UNC-0379. Four days after treatment, the percentage of plasma cells was measured by flow cytometry after staining with anti-CD138 and anti-CD38 antibodies. As shown in Fig. 2c, the viability of normal mature plasma cells remained roughly unaffected by UNC-0379 treatment except at the highest dose for one donor. In contrast, the median number of malignant plasma cells in MM patient samples was decreased in a dose-dependent manner with 50% and 63% of reduction with 2.5 µM and 5 µM of UNC-0379 respectively (*p* = 0.001 and *p* < 0.0001; *N* = 8), whereas non-tumoral CD138-negative BM cells displayed less sensitivity, like normal plasma cells (Fig. 2d). Consistent with a cytotoxicity of UNC-0379 preferentially in malignant plasma cells, normal plasma cells generated in vitro by differentiation of memory B cells remained largely unaffected by SETD8 inhibitor, even at high doses (Additional files 2 and 3: figure S5). In the perspective of potential preclinical studies with SETD8 inhibitors, primary 5T33vv murine MM models [25] were also treated with growing concentrations of UNC-0379 for 24 hours before cell viability analysis. As observed with human primary MM cells, UNC-0379 treatment results in a significant reduction of 5T33vv viability in a dose dependent manner (Additional files 2 and 3: Figure S6). Altogether, these results indicate that malignant plasma cells are particularly addicted to SETD8 activity and that UNC-0379 treatment is highly toxic to these tumor cells, leading rapidly to their growth inhibition and death.

### SETD8 inhibition impairs MM cell proliferation together with activation of p53-target gene pathways

**Fig. 3:**
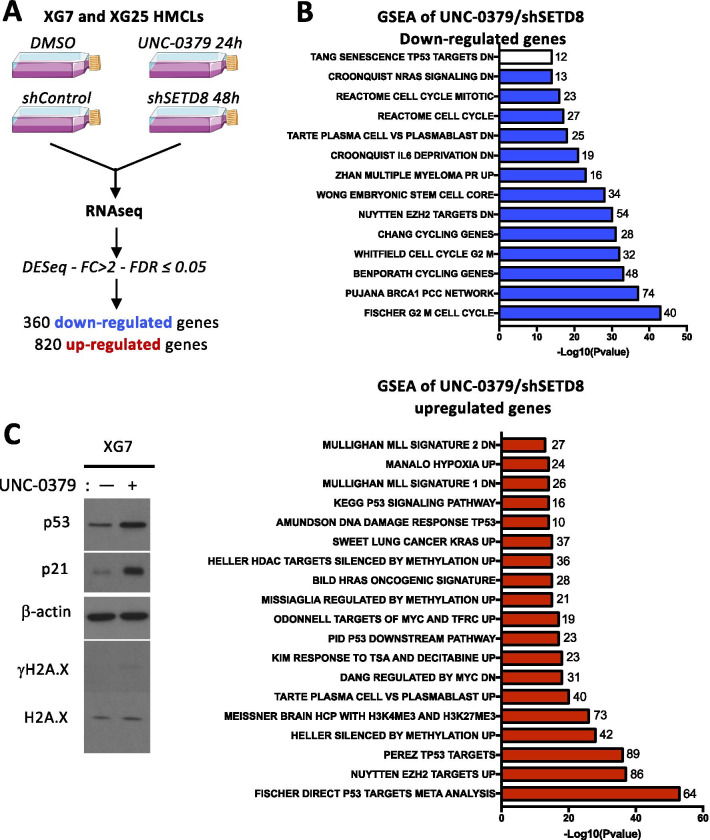
Gene expression changes in HMCLs upon genetic or pharmacological inhibition of SETD8. **a** Diagram of the experimental plan. **b** Molecular signatures of UNC-0379 and shSETD8 downregulated (blue bar plot) and up-regulated (red bar plot) genes compared to control were investigated using GSEA Database (all curated gene sets), and relevant pathways are presented (FDR *q* value ≤ 0.05). **c** Immunoblot analysis of indicated proteins in total lysates from UNC-0379-treated or untreated XG7 HMCL. β-actin and H2A.X were used as loading controls

SETD8 has been involved in all nuclear processes that use DNA as a matrix, including a critical role in the regulation of gene expression [8]. In order to gain insights into the mechanisms contributing to SETD8-mediated MM cell growth inhibition and death, we performed transcriptome analysis in XG7 and XG25 HMCLs after SETD8 inhibition using RNA sequencing (Fig. 3a). To identify gene expression alterations caused by SETD8 inhibition in HMCLs, we isolated total RNAs 18 h after UNC-0379 treatment or 48 h after shRNA-induced SETD8 silencing, when H4K20me1 decrease had not yet triggered DNA replication defects [31, 43]. A common signature of 820 up-regulated genes and 360 down-regulated genes was found in UNC-0379-treated and SETD8-depleted cell lines compared to the untreated and shRNA control cell lines (Fold change > 2; FDR ≤ 0.05) (Additional files 2 and 3: Figure S7A and Additional file 1: Table 1)*. Gene Set Enrichment Analysis* (GSEA) identified, as the most down-regulated pathways, genes involved in cell-cycle, stem cells, proliferating plasmablasts and MM proliferating molecular subgroup together with genes repressed upon loss of the histone H3K27 methyltransferase EZH2 (Figs. 3b and Additional files 2 and 3: Figure S7B). Conversely and consistent with the presence of a functional p53 in XG7 and XG25 cell lines [41], a significant positive enrichment was found for p53 target genes, including *p21* and *GADD45A* genes, and genes overexpressed in mature BMPCs versus plasmablasts (Fig. 3b and Additional files 2 and 3: Figure S7C). A positive enrichment for genes normally down-regulated by c-MYC, EZH2, DNA methylation or histone deacetylases (HDAC) was also observed (Fig. 3b), which might suggest some impairment of chromatin silencing pathways upon SETD8 inhibition in MM cells. As shown in Fig. 3c, immunoblot analysis of UNC-0379-treated XG7 HMCLs confirmed the increased levels of p53 and p21 proteins upon UNC-0379 treatment. However, we did not observe a significant phosphorylation of the histone variant H2A.X (Fig. 3c), suggesting p53 activation in XG7 MM cells treated with UNC-0379 likely occurs in absence or low level of DNA damage. Altogether, these data indicate that the cytotoxic effects of SETD8 inhibition in p53-proficient HMCLs are associated with activation of the p53 canonical pathway and a mature non-proliferating plasma cell transcriptional signature.

### The cytotoxicity of UNC-0379 treatment is not dependent on p53 in malignant plasma cells

**Fig. 4:**
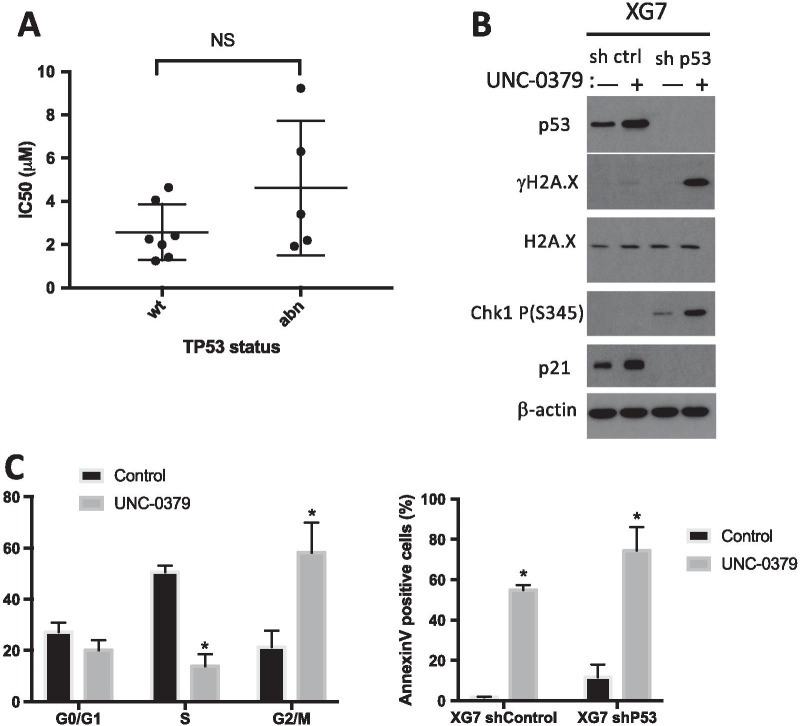
UNC-0379 toxicity is independent of p53. **a** Comparison of UNC-0379 IC_50_ (µM) according to HMCLs *TP53* status. **b** Immunoblot analysis of indicated proteins in total lysates from UNC-0379-treated (5 µM) or untreated XG7-shControl and XG7-shp53 HMCLs. β-actin and H2A.X were used as loading controls. **c** First bar plot represents cell cycle of control and 48 h UNC-0379-treated (5 µM) XG7-shp53 analyzed by flow cytometry using DAPI, BrdU incorporation and labelling with an anti-BrdU antibody. Results are representative of three independent experiments. Second bar plot represents apoptosis induction in control and UNC-0379-treated (5 µM) XG7-shControl and XG7-shp53 analyzed by AnnexinV-PE staining and flow cytometry after 96 h treatment. Data shown are mean values ± SD of 4 separate experiments. Statistical analysis was done with a paired *t* test. *Indicates a significant difference compared to control cells using a Wilcoxon test for pairs (*p* ≤ 0.05)

Previous studies showed that UNC-0379-induced cell death depends on the activation of p53 in neuroblastoma cancer models (NB), thereby rending p53-deficient NB cells more resistant to SETD8 inhibition [41]. To determine whether the cytotoxic effects of the pharmacological inhibition of SEDT8 mediated by UNC-0379 were also dependent on p53 activation in MM cells, we compared the UNC-0379 response in p53 wild-type HMCLs (*n* = 7) and p53-deficient HMCLs (*n* = 5) [28, 41] that displayed similar *SETD8* expression levels (Additional files 2 and 3: figure S8A). Of note, there was no significant correlation between *SETD8* and *TP53* expression in HMCLs (Additional files 2 and 3: Figure S8B). Although IC_50_ values for p53-deficient HMCLs following UNC-0379 treatment were more heterogeneous, we found no statistical difference with the group of p53 wild-type HMCLs (Fig. 4a). We also observed no statistical difference in the IC_50_ values of UNC-0379 according to the expression levels of p53 in HMCLs, whatever their mutational p53 status (Additional files 2 and 3: Figure S9). Altogether, these results suggested that the absence of a functional p53 did not alter significantly the sensitivity of malignant plasma cells to the pharmacological inhibition of SETD8 activity. To verify this hypothesis, we examined by immunoblot and flow cytometry analysis the cytotoxic effects of UNC-0379 treatment on p53-proficient XG7 HMCL transduced with high-titer of lentivirus encoding either a p53 shRNA or an irrelevant control shRNA. Both cell lines showed relatively similar IC_50_ values for UNC-0379 (3.7 µM in control shRNA and 4.8 µM in p53 shRNA expressing cells). Immunoblot analysis confirmed the efficient p53 depletion in p53 shRNA XG7 HMCL compared to control cells (Fig. 4b). Consistent with results in Fig. 3c, UNC-0379 treatment led to an up-regulation of p53 without detectable DNA damage in shRNA control XG7 cells (Fig. 4b). In contrast, higher levels of DNA damage and replicative stress were observed in UNC-0379-treated XG7 cells depleted for p53, as evidenced by the increased levels of both phosphorylated histone variant H2A.X and checkpoint protein Chk1 (Fig. 4b) and by an accumulation of cells in G2/M phase of the cell cycle (Fig. 4c)*.* Accordingly, an increase in DNA damage and replicative stress was also observed in XG1 HMCL naturally harboring a mutated inactive p53 (Additional files 2 and 3: Figure S10A). In XG1, as well as in XG7 inactive for p53, this was followed by changes in cell cycle distribution (Additional files 2 and 3: Figure S10B) and the appearance of a high percentage of apoptotic cells (Fig. 4c and Additional files 2 and 3: Figure S10C). Thus, these results indicated that UNC-0379-induced cytotoxicity in MM cells is independent on p53 status and that a high level of SETD8 likely protects MM cells from DNA damage and excessive replicative stress.

### UNC-0379 induces nucleolar stress independently of p53 activation

**Fig. 5:**
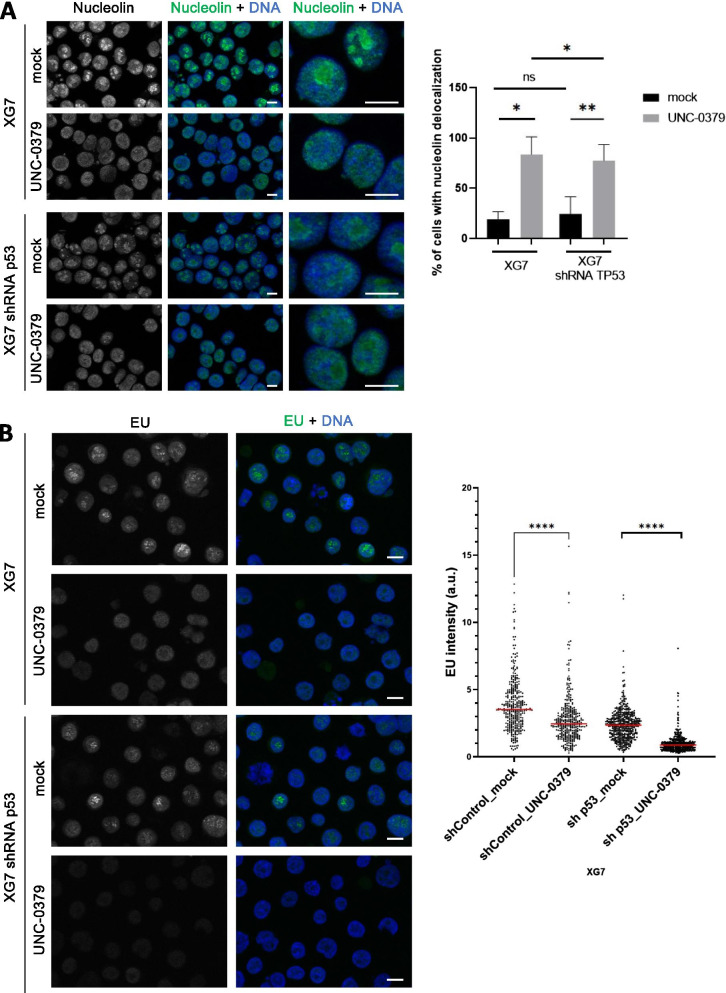
UNC-0379 treatment causes similar nucleolar alterations in wild-type and p53 depleted-myeloma cells. **a** Immunofluorescence microscopy of nucleolin localization and DNA (DAPI staining) in XG7 cells depleted or not for p53 and treated with 3 μM of UNC-0379 or the vehicle DMSO (mock) for 24 h. Scale bar: 10 μm. Visual quantification of percentage of cells with nucleolin diffusion out of the nucleolus. A minimum of 300 cells were quantified per condition. *N* = 3. Statistical significance between conditions was assessed using Student paired *t* test (*p value < 0.05, **p value < 0.01, ns: not significative). **b** Analysis of RNA synthesis by 5 min 5-Ethynyl Uridine (EU) pulse-labeling in XG7 cells depleted or not for p53 and treated with 3 μM of UNC-0379 or the vehicle DMSO (mock) for 24 h. Scale bar: 10 μm. EU intensity was quantified using CellProfiler “Human cell” pipeline. A minimum of 200 cells were quantified per condition. Graph shows the quantification of one representative experiment of 3

Nucleolar stress can lead to cell-cycle defects and apoptosis with and without p53 [44]. Since SETD8 interacts with nucleolar proteins and has been involved in the regulation of nucleoli structure and function [45, 46], we asked whether the cytotoxicity of UNC-0379 treatment might be related to nucleolar dysfunction independently of p53 activation in myeloma cells. To address this question, we first examined, by immunofluorescence, the localization of nucleolar proteins fibrillarin and nucleolin in control and p53-depleted XG7 cells untreated or treated with 3 μM of UNC-0379 for 24 h. Whereas the steady-state levels of nucleolar proteins and the localization of fibrillarin remained largely unaltered in all experimental conditions (Additional files 2 and 3: figure S11), a massive delocalization of the nucleolin pool from nucleoli to the nucleoplasm was observed in control and p53-depleted XG7 cells upon UNC-0379 treatment (Fig. 5a). Nucleolin is required for the transcription of ribosome DNA (rDNA) and alteration of its nucleolar transcriptional function can lead to apoptosis in cancer cells [47]. Therefore, to determine whether nucleolin delocalization upon UNC-0379 is associated with defective nucleolar transcription, cells were pulsed labeled with 5-Ethynyl Uridine (EU) for 5 min and nascent RNAs incorporating EU were detected in situ by click chemistry. Consistent with nucleoli being the sites of the most active RNA synthesis, short EU pulse labeling resulted in an intense granular staining specifically in nucleoli of untreated control and p53-depleted XG7 cells (Fig. 5b). Upon UNC-0379 treatment, this intense nucleolar EU staining was almost abolished in both control and p53 depleted cells, indicating a strong decrease of rDNA transcription independently of p53 activation. Taken together, these results show that, prior to p53 activation, treatment with UNC-0379 leads to specific nucleolar alterations characterized by massive nucleolin delocalization and a strong decrease in nucleolar RNA synthesis, thus providing an explanation for the cytotoxic effects of this SETD8 inhibitory compound on malignant plasma cells regardless of p53 status.

### UNC-0379-mediated inhibition of SETD8 synergizes with melphalan

**Fig. 6:**
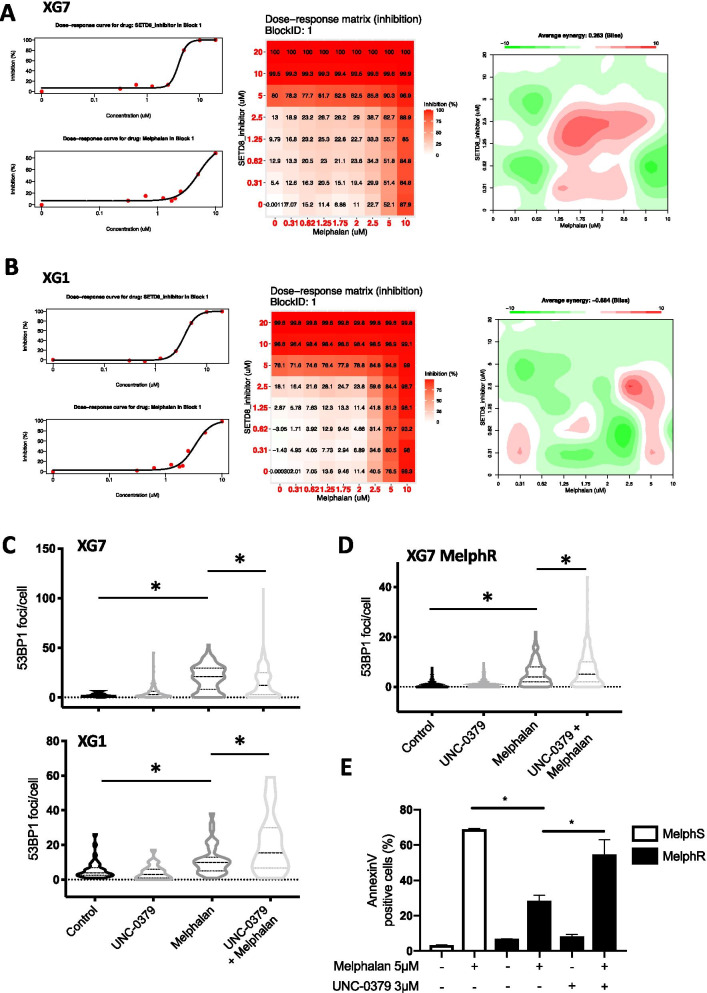
UNC-0379 treatment sensitizes HMCLs to Melphalan-induced DNA damage. **a** Dose–response matrix inhibition and landscape representation for experimentally measured drug synergy in XG7 cells treated with increased concentration of the combination of melphalan and SETD8 inhibitor (UNC-0379). Synergy scores are displayed using a continuous pseudo-color scale ranging from dark-green = antagonistic to dark-red = synergistic **b** Landscape and 3-D matrix representation for experimentally measured drug synergy in XG1 cells. **c** Number of 53BP1 foci in XG7 and XG1 HMCLs, treated with melphalan (3 µM), UNC-0379 (3 µM) or the combination of the two drugs for 6 h or 24 h as indicated. Number of foci per cell was quantified using ImageJ software (mean number of cells counted: 300). Statistical significance between conditions was assessed using Student paired *t* test (*p value < 0.05). **d** Measure of 53BP1 foci in melphalan-resistant XG7 cells treated with melphalan (15 µM), UNC-0379 (3 µM) or the combination of the two drugs for 24 h. Number of foci per cell was quantified using ImageJ software (mean number of cells counted: 300). Statistical significance between conditions was assessed using Student paired *t* test (*p value < 0.05). **e** Measure of AnnexinV-positive cells in the same drug concentrations as in D. AnnexinV-PE staining was analyzed by flow cytometry 96 h after drug treatment. Statistical significance between conditions was assessed using Student paired *t* test (*p value < 0.05)

**Fig. 7:**
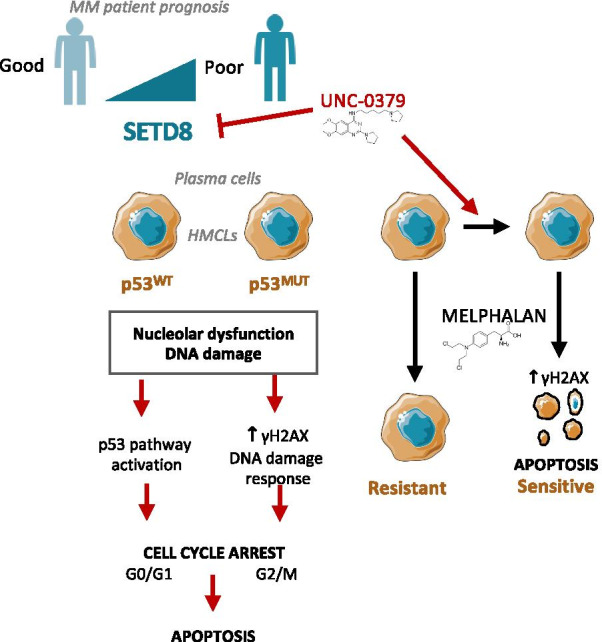
SETD8 inhibition is of therapeutic interest in MM independently of p53 status and could overcome melphalan resistance. High *SETD8* expression is associated with a poor outcome in MM and represent a therapeutic target independently of p53 status. In p53 WT patients SETD8 inhibition results in MM cell cytotoxicity mediated by p53 pathway activation. In p53 deficient MM cells, SETD8 inhibition increases replicative stress in association with MM cell death. Furthermore, SETD8 inhibition overcomes melphalan resistance in MM

The results presented above indicate that the pharmacological inhibition of SETD8 by UNC-0379 could constitute a promising strategy to improve MM treatment by genotoxic agents. To gain further insights into this possibility, we investigated whether SETD8 inhibition could enhance the cytotoxicity of melphalan and overcome resistance to this alkylating agent widely used in MM treatment. To this end, we first measured the viability of XG7 and XG1 cells continuously treated for 4 days with different concentrations of each compound alone or in combination. The synergy score of each combination dose was then evaluated using the Bliss model. As shown in Fig. 6a, b, we observed a significant synergism of the combination compared to the individual treatments in XG7 cell line and, to a lesser extent, in XG1 for the highest melphalan doses (Fig. 6a, b). Importantly, the higher sensitivity to melphalan was also observed in XG7 cells depleted for SETD8 after lentiviral shRNA expression (Additional files 2 and 3: figure S12A-C) and, consistent with results in Fig. 4c, XG7 cells depleted for p53 displayed a similar sensitivity to the combination than the shRNA-control XG7 cells (Additional files 2 and 3: Figure S12D). These data demonstrate that the lethal synergy between UNC-0379 and melphalan is indeed related to SETD8 inhibition and does not depend on p53 activation. As shown in Fig. 6c, the lethality induced by the co-treatment of melphalan and UNC-0379 was preceded by high levels of DNA breaks, as measured by the significant increase in the number of DNA damage-induced 53BP1 foci in combined versus single agent therapy, in both XG7 and XG1 cell lines. This was also observed in melphalan-resistant XG7 clones [29] (Fig. 6d), which coincided with higher levels of γH2AX signal upon melphalan/UNC-0379 combination (Additional files 2 and 3: Figure S13). At later time points, we observed a significant increase in apoptotic cell death as measured by annexin V staining (Fig. 6e). Altogether, these results indicate that UNC-0379-mediated SETD8 inhibition enhances the cytotoxic effects of melphalan in MM cells in a p53 independent manner. This demonstrates the potential therapeutic interest to target SETD8-mediated lysine methylation in MM regardless of p53 status (Fig. 7).

## Discussion

This study unveils for the first time a role of the histone H4K20 mono-methyltransferase SETD8 in MM pathophysiology. We have shown that elevated levels of SETD8 are associated with a poor prognosis and correlate with the deregulation of regulatory nodes involved in MM, including IRF4, MYC/MAX, MAPK and DNA repair pathways (Fig. 1 and Additional files 2 and 3: Figure S2). Moreover, patients displaying higher SETD8 expression are associated with specific molecular features, such as increased copy of Chr1q21 or a high GPI index (Additional files 2 and 3: Figure S1). Consistent with this, primary MM cells are particularly addicted to SETD8 activity and the recently developed small-molecule inhibitor of SETD8, UNC-0379, demonstrated a higher toxicity in MM cells compared to normal cells from the bone marrow microenvironment and normal plasma cells (Fig. 2). From a mechanistic point of view, our RNA-seq results show that genetic or pharmacological inhibition of SETD8 in MM cell lines results in the activation of a mature non-proliferating plasma cell signature and of the p53 canonical pathway (Fig. 3). However, UNC-0379-induced cytotoxicity does not necessarily require p53 activation as p53 wild-type and deficient MM cells display similar sensitivity to SETD8 inhibition (Figs. 3 and 4). Indeed, we provide evidence that UNC-0379 is an inhibitor of nucleolar RNA synthesis independently of p53 activation (Fig. 5). Finally, we have shown that UNC-0379 treatment leads to a significant lethal synergy with the DNA-damaging agent melphalan and can overcome the resistance associated with this cytotoxic drug in both p53 wild-type and deficient MM cells (Fig. 6). Altogether, these results provide evidence for SETD8 inhibition as a potential novel therapeutic strategy in MM independently from the mutational p53 status of these tumors (Fig. 7).

An up-regulation of SETD8 is not specific to MM, as it has also been observed in different types of solid tumors [11], such as papillary thyroid cancer [16], breast carcinoma [14, 48], and childhood tumors of the nervous system [15, 18]. However, the mechanisms that contribute to elevated levels of SETD8 in cancer still remained unclear. In neuroblastoma, a higher level of SETD8 could be due in part to DNA copy-number gains at chr12q24, the region that encompasses *SETD8* encoding gene^26^. Another non-exclusive mechanism is related to the deregulation of oncogenic c-MYC pathway, since *SETD8* encoding gene has been found as a transcriptional target of c-MYC [49] and functionally required to mediate MYC-induced cell growth [15]. It is widely established that c-MYC is a key regulator in MM with deregulations related to translocations, gains and amplification, mutations in RAS genes and MYC transcription or translation activation [50]. Interestingly, while chr12q24 gains are not present in myeloma, we identified here a significant enrichment of c-MYC/MAX target genes in MM patients characterized by high *SETD8* expression, thereby supporting a potential role of this oncogenic pathway in SETD8 deregulation. Furthermore, SETD8 depletion or inhibition results in up-regulation of genes repressed upon c-MYC expression (Fig. 3c) suggesting that SETD8 might also participate in deregulation of c-MYC functions in MM.

A striking result of our study is that the up-regulation of SETD8 in malignant plasma cells is not associated with visible changes in the global levels of the histone H4K20 mono-methylation (H4K20me1), the primary target of SETD8 [8], although we cannot rule out that some local changes can occur at specific loci. This result is independent from cell-cycle progression, since changes in the levels of SETD8 do not correlate with cell proliferation (Fig. 1b and Additional files 2 and 3: Figure S2). One hypothesis is that the overexpression of the H4K20me1-specific demethylase PHF8, observed in many cancers including hematopoietic malignancies [51, 52], could attenuate the impact of SETD8 up-regulation on histone H4K20 [53]. Nevertheless, H4K20me1 is critical in the maintenance of genome integrity [8] and its decrease upon UNC-0379 treatment would most likely contribute to the cytotoxic effects of this SETD8 inhibitor in myeloma cells.

Recent studies have suggested that the role of SETD8 in cancer might also involve the methylation of other substrates than histone H4. Thus, Takawa et al. proposed that SEDT8 can methylate the proliferating cell nuclear antigen PCNA and thus favor HeLa cell proliferation [11]; but this result was never further confirmed in other cell types. Additionally, SETD8 can induce the mono-methylation of the tumor suppressor p53 at lysine 382 (p53K382me1), which attenuates its pro-apoptotic and growth arrest functions [12, 18]. Hence, in neuroblastoma, inhibition of SETD8 by UNC-0379 leads to cell death in a p53-dependent manner and p53K382me1 is important for this phenotype [18]. Our RNA-seq and functional studies in MM cells show that the genetic or pharmacological inhibition of SETD8 leads to the activation of p53 conventional pathways, which correlates with an increased in p53 and p21 protein levels, and cell cycle arrest followed by cell death. However, in contrast to neuroblastoma [18], UNC-0379-induced cytotoxicity in MM cells is not necessarily dependent on p53 activation and, despite several attempts, we were unable to detect p53K382me1. Since the appearance of nucleolar stress or DNA damage is sufficient to activate p53 functions [54, 55] and occurs independently of p53 upon UNC-0379 (Figs. 4 and 5), we propose that the activation of p53 upon SETD8 inhibition is mainly caused by these stress signals rather than the potential loss of SETD8-induced p53 methylation in MM cells. Since nucleolar dysfunction can lead to apoptosis without p53 [44], the induction of these cellular stress prior to p53 activation provides an explanation of the cell-growth inhibition and apoptotic effects of UNC-0379 in p53-deficient cells. While the appearance of DNA damage might be related to SETD8 functions in chromatin structure [50], the reasons of nucleolar alterations upon UNC-0379-mediated SETD8 inhibition remain unclear. Future work will be necessary to unravel the SETD8 nucleolar functions, which could be related to the apparent ability of SETD8 to interact directly with ribosomal proteins or regulate their expression [45, 46]. Altogether, our findings are consistent with a model in which UNC-0379-mediated cell death is triggered by multiple cellular stress signals followed by p53 activation in p53-proficient MM cells and deadly cell-cycle progression in absence of p53, thereby rending multiple myeloma sensitive to SEDT8 inhibition whatever the status of p53.

In spite of effective therapeutic protocols developed in MM, drug resistance remains a major concern. In this regard, we report here that high *SETD8* expression is associated with a poor prognosis in patients treated by high-dose of melphalan and autologous stem cell transplantation (Fig. 1). SETD8 expression is also significantly up-regulated in patients at relapse compared to newly diagnosed patients (Additional files 2 and 3: Figure S3). In agreement with this, UNC-0379 treatment potentiates the cytotoxicity of melphalan and overcomes melphalan drug resistance in MM cells, underlining the interest to target SETD8 to improve the treatment of MM patients.

Deletion of the short arm of chromosome 17 (del 17p) is associated with a poor outcome in MM independently of treatment regimen [56–60]. Interestingly, MM cell toxicity mediated by SETD8 inhibitor is mainly p53-independent. The frequency of events targeting p53 (del 17p, *TP53* mutation or double hits) increases during the progression of MM and consecutive relapses underlining a selection of cells harboring *TP53* abnormalities in association with resistance to treatment. *TP53* bi-allelic events are also associated with a dramatic impact on MM patients’ survival after relapse [61]. Therapies inducing significant toxicity of p53 defective-MM cells are needed. Our results demonstrate the therapeutic interest of SETD8 inhibitor to target p53 deficient MM cells by increasing replicative stress and DNA breaks. Accordingly, the use of this epigenetic drug in combination with cytotoxic agents, such as melphalan, could be of clinical interest, notably in newly diagnosed patients presenting del17p and/or *SETD8* overexpression and eligible to high dose melphalan and ASCT (Fig. 6). Thus, SETD8 inhibition appears of therapeutic interest to overcome drug resistance and improve the treatment of MM patients at relapse independently of the p53 status.

## Conclusion

Our data reveal that the up-regulation of the histone H4K20 mono-methyltransferase SETD8 is associated with a poor prognosis and the deregulation of major signaling pathways in multiple myeloma patients. The inhibition of this enzyme leads to deadly cellular stress in multiple myeloma cells and a lethal synergy was identified when SETD8 inhibition is combined with melphalan alkylating agent. Altogether, these results demonstrate that SETD8 is a novel epigenetic target in multiple myeloma and that its pharmacological inhibition could be beneficial in high-risk MM patients whatever their p53 status.

## Supplementary Information

**Additional file 1: Table 1 Genes regulated by SETD8 in p53wt myeloma cell lines.** Differential expression analysis was performed using DESeq2 pipeline. p values were adjusted to control the global FDR across all comparisons with the default option of the DESeq2 package. Genes were considered differentially expressed if they had an adjusted p value of 0.05 and a fold change of 1.5.

**Additional file 2**. Legends of supplementary Figures.

**Additional file 3. Supplementary Figure S1:** SETD8 expression status in MM patients (**A**) Gene expression profiling of MMCs of the patients of UAMS-TT2 cohort were used. PR: proliferation, LB: low bone disease, MS: MMSET, HY: hyperdiploid, CD1: Cyclin D1-Cyclin D3, CD2: Cyclin D1-Cyclin D3, MF: MAF, MY: myeloid. (**B**) SETD8 expression in 186 patients of the UAMS-TT2 cohort showing Ch1q21 copy number aberration. (**C**) SETD8 expression in MMCs (patients at diagnosis) presenting low, medium or high gene expression-based proliferation index (GPI). (**D**) Correlation between SETD8 expression and malignant plasma cell labeling index. Plasma cell labeling index was investigated using BrdU incorporation and flow cytometry in 101 patients at diagnosis. **Supplementary Figure S2**: Gene Signature of MM patients with high SETD8 expression. GSEA enrichment plots with the absolute enrichment p value and the normalized enrichment score of the gene set. **Supplementary Figure S3**: SETD8 is more expressed in patients at relapse compared to diagnosis. Boxplot showing SETD8 expression in MM cells of patients at relapse (n=47) compared to diagnosis (n=205). **Supplementary Figure S4**: UNC-0379 inhibits SETD8 and affects MM cells survival and cell cycle. (**A**) Immunoblot analysis of SETD8, Histone H4 and H4K20me1 protein levels in XG7 and XG25 cells untreated or treated with 3 μM of UNC-0379 for 24 hours. (**B**) Quantitation of cell-cycle distribution of control (untreated) and UNC-0379-treated XG7 and XG25 HMCLs 48 hours after treatment. After short-pulse of BrdU incorporation, cell-cycle was analyzed by FACS using DAPI and anti-BrdU antibody. (**C**) Quantitation of apoptosis in control and UNC-0379-treated XG7 and XG25 HMCLs by flow cytometry with AnnexinV-PE staining and 96h after UNC-0379 treatment. Data shown are mean values ± SD of 4 separate experiments. Statistical analysis was done with a paired t-test. (*) indicates a significant difference compared to control cells using a Wilcoxon test for pairs (P ≤ 0.05). **Supplementary Figure S5**: UNC-0379 treatment did not affect in vitro differentiation of human memory B cells. In vitro differentiation of memory B cells into plasma cells from 2 different donors. Cells were treated at Day 7 with UNC-0379 at the indicated concentrations and analyzed at Day 10. (**A**) Total cell number was visually quantified. Dead cells were visualized by trypan blue staining and viability was calculated as the ratio of living cells vs total cells. Annexin V+ cells were quantified by FACS. (**B**) Percentage of each population at day 10 (MBC: memory B cells, PrePB: preplasmablasts, PB: plasmablasts, PC: plasma cells). Mock is treated with the same percentage of DMSO as 5 μM sample. **Supplementary Figure S6**: UNC-0379 affects murine MM cells viability. Measure of viability and apoptosis in primary murine 5T3vv cellular models untreated (cnt) or treated with growing concentrations (from 2.5 to 40 μM) of UNC0379 for 24 hours. **Supplementary Figure S7**: UNC-0379 treatment deregulates gene expression in HMCLs. (**A**) Heatmap of RNA-sequencing rlog expression data. Genes deregulated by UNC-0379 and shSETD8 in XG7 and XG25 HMCLs are represented. Expression scale shows low expression levels in blue and high expression levels in red. (**B**) Bar-plot representing the fold expression (UNC-0379 condition over control) of genes related to Fig 1B chart pathways. (**C**) Bar-plot representing the fold expression (UNC-0379 condition over control) of genes related to Fig 1B chart pathways. **Supplementary Figure S8**: SETD8 expression correlation with p53 expression or status. (**A**) Comparison of SETD8 expression according to HMCLs TP53 status. (**B**) Correlation between TP53 and SETD8 expression in HMCLs. TP53 and SETD8 expression was obtained from Affymetrix microarrays data previously published. **Supplementary Figure S9**: Correlation between TP53 expression and drug response to UNC-0379 in HMCLs. IC50 was determined using CTG-based growth assay, TP53 expression was obtained from Affymetrix microarrays data previously published. **Supplementary Figure S10**: UNC-0379 induces genomic instability in XG1 p53-mutant cell line. (**A**) Immunoblot analysis of indicated proteins in total lysates from UNC-0379-treated (5µM) or untreated XG1 HMCL. β-actin and H2A.X were used as loading controls. (**B**) Cell cycle of control and 48h UNC-0379-treated (5µM) XG1 HMCL was analyzed by flow cytometry using DAPI, BrdU incorporation and labelling with an anti-BrdU antibody. Results are representative of three independent experiments. * indicates a significant difference compared to control cells using a Wilcoxon test for pairs (P ≤ 0.05). (**C**) Apoptosis induction was investigated using AnnexinV-PE staining by flow cytometry. **Supplementary Figure S11**: UNC-0379 did not alter the steady states levels of nucleolar proteins and the nucleolar localization of fibrillarin. (**A**) immunoblot analysis of indicated proteins in shRNA control and p53-depleted XG7 cells treated with the vehicle DMSO or 3 μM of UNC-0379 for 24 hours. Ponceau staining is used as loading control (**B**) immunofluorescence microscopy of fibrillarin localization and DNA (DAPI staining) in XG7 cells depleted or not for p53 at low and higher magnitude as indicated. Scale bar : 10 µM. **Supplementary Figure S12**: The lethal synergy between UNC-0379 and melphalan is caused by SETD8 inhibition but does not depend on p53 activation. (**A**) immunoblot analysis of SETD8 and tubulin protein levels in total lysates of XG7 cells expressing a doxycycline-inducible shRNA targeting SETD8 mRNA and treated or not with doxycycline (1µg/ml) for 4 days. (**B**) The graph shows one representative from 3 independent experiments. Cell proliferation analysis was performed by CTG, 4 days after co-treatment with Doxycycline (1ug/ml) to silence SETD8 and various concentrations of melphalan as indicated. (**C**) IC50 values from 3 independent experiments performed as in (**B**). p-value = 0.0196. (**D**) Measure by flow cytometry of apoptosis induction (AnnexinV-PE staining) in XG7-shControl and XG7-shTP53 HMCLs after 96h of treatment with Melphalan (5μM), UNC-0379-treated (3μM) or the combination of the two drugs. **Supplementary Figure S13**: Immunoblot analysis of histone H2AX and its phosphorylated form γH2A.X (marker of DNA damage) in total lysates from melphalan-resistant XG7 HMCLs. Cells were treated with melphalan (5μM), UNC-0379-treated (3μM) or the combination of the two drugs for 24h. β-actin was used as loading control.

## References

[1] Palumbo A, Anderson K (2011). Multiple myeloma. N Engl J Med.

[2] Siegel RL, Miller KD, Jemal A (2018). Cancer statistics, 2018. CA Cancer J Clin.

[3] Multiple myeloma, (2018). update on diagnosis, risk-stratification, and management. Am J Hematol.

[4] De Smedt E, Lui H, Maes K, De Veirman K, Menu E, Vanderkerken K (2018). The epigenome in multiple myeloma: impact on tumor cell plasticity and drug response. Front Oncol.

[5] Herviou L, Kassambara A, Boireau S, Robert N, Requirand G, Müller-Tidow C (2018). PRC2 targeting is a therapeutic strategy for EZ score defined high-risk multiple myeloma patients and overcome resistance to IMiDs. Clin Epigenetics.

[6] Bruyer A, Maes K, Herviou L, Kassambara A, Seckinger A, Cartron G (2018). DNMTi/HDACi combined epigenetic targeted treatment induces reprogramming of myeloma cells in the direction of normal plasma cells. Br J Cancer.

[7] Sivaraj D, Green MM, Gasparetto C (2017). Panobinostat for the management of multiple myeloma. Future Oncol Lond Engl.

[8] Beck DB, Oda H, Shen SS, Reinberg D (2012). PR-Set7 and H4K20me1: at the crossroads of genome integrity, cell cycle, chromosome condensation, and transcription. Genes Dev.

[9] Brustel J, Tardat M, Kirsh O, Grimaud C, Julien E (2011). Coupling mitosis to DNA replication: the emerging role of the histone H4-lysine 20 methyltransferase PR-Set7. Trends Cell Biol.

[10] Jørgensen S, Schotta G, Sørensen CS (2013). Histone H4 lysine 20 methylation: key player in epigenetic regulation of genomic integrity. Nucleic Acids Res.

[11] Takawa M, Cho H-S, Hayami S, Toyokawa G, Kogure M, Yamane Y (2012). Histone lysine methyltransferase SETD8 promotes carcinogenesis by deregulating PCNA expression. Cancer Res.

[12] Shi X, Kachirskaia I, Yamaguchi H, West LE, Wen H, Wang EW (2007). Modulation of p53 function by SET8-mediated methylation at lysine 382. Mol Cell.

[13] Dhami GK, Liu H, Galka M, Voss C, Wei R, Muranko K (2013). Dynamic methylation of Numb by Set8 regulates its binding to p53 and apoptosis. Mol Cell.

[14] Huang R, Yu Y, Zong X, Li X, Ma L, Zheng Q (2017). Monomethyltransferase SETD8 regulates breast cancer metabolism via stabilizing hypoxia-inducible factor 1α. Cancer Lett.

[15] Veo B, Danis E, Pierce A, Sola I, Wang D, Foreman NK, et al. Combined functional genomic and chemical screens identify SETD8 as a therapeutic target in MYC-driven medulloblastoma. JCI Insight. 2019;4. 10.1172/jci.insight.122933 PMC6485357 30626740

[16] Liao T, Wang Y-J, Hu J-Q, Wang Y, Han L-T, Ma B (2018). Histone methyltransferase KMT5A gene modulates oncogenesis and lipid metabolism of papillary thyroid cancer in vitro. Oncol Rep.

[17] Hou L, Li Q, Yu Y, Li M, Zhang D (2016). SET8 induces epithelial-mesenchymal transition and enhances prostate cancer cell metastasis by cooperating with ZEB1. Mol Med Rep.

[18] Veschi V, Liu Z, Voss TC, Ozbun L, Gryder B, Yan C (2017). Epigenetic siRNA and chemical screens identify SETD8 inhibition as a therapeutic strategy for p53 activation in high-risk neuroblastoma. Cancer Cell.

[19] Teoh PJ, Chng WJ (2014). p53 abnormalities and potential therapeutic targeting in multiple myeloma. BioMed Res Int.

[20] Hose D, Moreaux J, Meissner T, Seckinger A, Goldschmidt H, Benner A (2009). Induction of angiogenesis by normal and malignant plasma cells. Blood.

[21] Hose D, Rème T, Meissner T, Moreaux J, Seckinger A, Lewis J (2009). Inhibition of aurora kinases for tailored risk-adapted treatment of multiple myeloma. Blood.

[22] Mulligan G, Mitsiades C, Bryant B, Zhan F, Chng WJ, Roels S (2007). Gene expression profiling and correlation with outcome in clinical trials of the proteasome inhibitor bortezomib. Blood.

[23] Barlogie B, Tricot G, Rasmussen E, Anaissie E, van Rhee F, Zangari M (2006). Total therapy 2 without thalidomide in comparison with total therapy 1: role of intensified induction and posttransplantation consolidation therapies. Blood.

[24] Pineda-Roman M, Zangari M, van Rhee F, Anaissie E, Szymonifka J, Hoering A (2008). VTD combination therapy with bortezomib-thalidomide-dexamethasone is highly effective in advanced and refractory multiple myeloma. Leukemia.

[25] Asosingh K, Günthert U, Bakkus MH, De Raeve H, Goes E, Van Riet I (2000). In vivo induction of insulin-like growth factor-I receptor and CD44v6 confers homing and adhesion to murine multiple myeloma cells. Cancer Res.

[26] De Bruyne E, Bos TJ, Asosingh K, Vande Broek I, Menu E, Van Valckenborgh E (2008). Epigenetic silencing of the tetraspanin CD9 during disease progression in multiple myeloma cells and correlation with survival. Clin Cancer Res Off J Am Assoc Cancer Res.

[27] Moreaux J, Reme T, Leonard W, Veyrune J-L, Requirand G, Goldschmidt H (2013). Gene expression-based prediction of myeloma cell sensitivity to histone deacetylase inhibitors. Br J Cancer.

[28] Moreaux J, Klein B, Bataille R, Descamps G, Maïga S, Hose D (2011). A high-risk signature for patients with multiple myeloma established from the molecular classification of human myeloma cell lines. Haematologica.

[29] de Boussac H, Bruyer A, Jourdan M, Maes A, Robert N, Gourzones C (2020). Kinome expression profiling to target new therapeutic avenues in multiple myeloma. Haematologica.

[30] Viziteu E, Klein B, Basbous J, Lin Y-L, Hirtz C, Gourzones C (2017). RECQ1 helicase is involved in replication stress survival and drug resistance in multiple myeloma. Leukemia.

[31] Tardat M, Murr R, Herceg Z, Sardet C, Julien E (2007). PR-Set7-dependent lysine methylation ensures genome replication and stability through S phase. J Cell Biol.

[32] Dobin A, Davis CA, Schlesinger F, Drenkow J, Zaleski C, Jha S (2013). STAR: ultrafast universal RNA-seq aligner. Bioinforma Oxf Engl.

[33] Gentleman RC, Carey VJ, Bates DM, Bolstad B, Dettling M, Dudoit S (2004). Bioconductor: open software development for computational biology and bioinformatics. Genome Biol.

[34] Love MI, Huber W, Anders S (2014). Moderated estimation of fold change and dispersion for RNA-seq data with DESeq2. Genome Biol.

[35] Liberzon A, Subramanian A, Pinchback R, Thorvaldsdóttir H, Tamayo P, Mesirov JP. Molecular signatures database (MSigDB) 3.0. Bioinforma Oxf Engl. 2011;27:1739–40. 10.1093/bioinformatics/btr260 PMC3106198 21546393

[36] Subramanian A, Tamayo P, Mootha VK, Mukherjee S, Ebert BL, Gillette MA (2005). Gene set enrichment analysis: a knowledge-based approach for interpreting genome-wide expression profiles. Proc Natl Acad Sci.

[37] Kassambara A, Rème T, Jourdan M, Fest T, Hose D, Tarte K (2015). GenomicScape: an easy-to-use web tool for gene expression data analysis. Application to investigate the molecular events in the differentiation of B cells into plasma cells. PLoS Comput Biol.

[38] Küffner R, Zach N, Norel R, Hawe J, Schoenfeld D, Wang L (2015). Crowdsourced analysis of clinical trial data to predict amyotrophic lateral sclerosis progression. Nat Biotechnol.

[39] Herviou L, Jourdan M, Martinez A-M, Cavalli G, Moreaux J (2019). EZH2 is overexpressed in transitional preplasmablasts and is involved in human plasma cell differentiation. Leukemia.

[40] Jourdan M, Caraux A, Caron G, Robert N, Fiol G, Reme T (2011). Characterization of a transitional preplasmablast population in the process of human B cell to plasma cell differentiation. J Immunol.

[41] Vikova V, Jourdan M, Robert N, Requirand G, Boireau S, Bruyer A (2019). Comprehensive characterization of the mutational landscape in multiple myeloma cell lines reveals potential drivers and pathways associated with tumor progression and drug resistance. Theranostics.

[42] Ma A, Yu W, Li F, Bleich RM, Herold JM, Butler KV (2014). Discovery of a selective, substrate-competitive inhibitor of the lysine methyltransferase SETD8. J Med Chem.

[43] Jørgensen S, Elvers I, Trelle MB, Menzel T, Eskildsen M, Jensen ON (2007). The histone methyltransferase SET8 is required for S-phase progression. J Cell Biol.

[44] James A, Wang Y, Raje H, Rosby R, DiMario P (2014). Nucleolar stress with and without p53. Nucleus.

[45] Tanaka H, Takebayashi S, Sakamoto A, Igata T, Nakatsu Y, Saitoh N (2017). The SETD8/PR-Set7 methyltransferase functions as a barrier to prevent senescence-associated metabolic remodeling. Cell Rep.

[46] Qin Y, Ouyang H, Liu J, Xie Y (2013). Proteome identification of proteins interacting with histone methyltransferase SET8. Acta Biochim Biophys Sin.

[47] Jia W, Yao Z, Zhao J, Guan Q, Gao L (2017). New perspectives of physiological and pathological functions of nucleolin (NCL). Life Sci.

[48] Yang F, Sun L, Li Q, Han X, Lei L, Zhang H (2012). SET8 promotes epithelial-mesenchymal transition and confers TWIST dual transcriptional activities. EMBO J.

[49] Driskell I, Oda H, Blanco S, Nascimento E, Humphreys P, Frye M (2012). The histone methyltransferase Setd8 acts in concert with c-Myc and is required to maintain skin. EMBO J.

[50] Jovanović KK, Roche-Lestienne C, Ghobrial IM, Facon T, Quesnel B, Manier S (2018). Targeting MYC in multiple myeloma. Leukemia.

[51] Björkman M, Östling P, Härmä V, Virtanen J, Mpindi J-P, Rantala J (2012). Systematic knockdown of epigenetic enzymes identifies a novel histone demethylase PHF8 overexpressed in prostate cancer with an impact on cell proliferation, migration and invasion. Oncogene.

[52] Fu Y, Yang Y, Wang X, Yin X, Zhou M, Wang S (2018). The histone demethylase PHF8 promotes adult acute lymphoblastic leukemia through interaction with the MEK/ERK signaling pathway. Biochem Biophys Res Commun.

[53] Liu W, Tanasa B, Tyurina OV, Zhou TY, Gassmann R, Liu WT (2010). PHF8 mediates histone H4 lysine 20 demethylation events involved in cell cycle progression. Nature.

[54] Huang LC, Clarkin KC, Wahl GM (1996). Sensitivity and selectivity of the DNA damage sensor responsible for activating p53-dependent G1 arrest. Proc Natl Acad Sci U S A.

[55] Loewer A, Karanam K, Mock C, Lahav G (2013). The p53 response in single cells is linearly correlated to the number of DNA breaks without a distinct threshold. BMC Biol.

[56] Munshi NC, Anderson KC, Bergsagel PL, Shaughnessy J, Palumbo A, Durie B (2011). Consensus recommendations for risk stratification in multiple myeloma: report of the international myeloma workshop consensus panel 2. Blood.

[57] Avet-Loiseau H, Leleu X, Roussel M, Moreau P, Guerin-Charbonnel C, Caillot D (2010). Bortezomib plus dexamethasone induction improves outcome of patients with t(4;14) myeloma but not outcome of patients with del(17p). J Clin Oncol Off J Am Soc Clin Oncol.

[58] Dimopoulos MA, Kastritis E, Christoulas D, Migkou M, Gavriatopoulou M, Gkotzamanidou M (2010). Treatment of patients with relapsed/refractory multiple myeloma with lenalidomide and dexamethasone with or without bortezomib: prospective evaluation of the impact of cytogenetic abnormalities and of previous therapies. Leukemia.

[59] Drach J, Ackermann J, Fritz E, Krömer E, Schuster R, Gisslinger H (1998). Presence of a p53 gene deletion in patients with multiple myeloma predicts for short survival after conventional-dose chemotherapy. Blood.

[60] Schilling G, Hansen T, Shimoni A, Zabelina T, Pérez-Simón J-A, Simon-Perez J-A (2008). Impact of genetic abnormalities on survival after allogeneic hematopoietic stem cell transplantation in multiple myeloma. Leukemia.

[61] Weinhold N, Ashby C, Rasche L, Chavan SS, Stein C, Stephens OW (2016). Clonal selection and double-hit events involving tumor suppressor genes underlie relapse in myeloma. Blood.

