# Supplementary material for: Targeting the methyltransferase SETD8 impairs tumor cell survival and overcomes drug resistance independently of p53 status in multiple myeloma
Source: Clin Epigenetics. 2021 Sep 16;13:174. doi: 10.1186/s13148-021-01160-z (PMC8447659; doi:10.1186/s13148-021-01160-z)
Supplement: Supplementary file 2 — Additional file 2. Legends of supplementary Figures. [file 13148_2021_1160_MOESM2_ESM.docx]

**Legends of supplementary Figures:**

**Supplementary Figure S1:** **SETD8 expression status in MM patients**

**(A)** Gene expression profiling of MMCs of the patients of UAMS-TT2 cohort were used. PR: proliferation, LB: low bone disease, MS: MMSET, HY: hyperdiploid, CD1: Cyclin D1-Cyclin D3, CD2: Cyclin D1-Cyclin D3, MF: MAF, MY: myeloid. **(B)** *SETD8* expression in 186 patients of the UAMS-TT2 cohort showing Ch1q21 copy number aberration. **(C)** *SETD8* expression in MMCs (patients at diagnosis) presenting low, medium or high gene expression-based proliferation index (GPI). **(D)** Correlation between SETD8 expression and malignant plasma cell labeling index. Plasma cell labeling index was investigated using BrdU incorporation and flow cytometry in 101 patients at diagnosis.

**Supplementary Figure S2: Gene Signature of MM patients with high SETD8 expression.** GSEA enrichment plots with the absolute enrichment p value and the normalized enrichment score of the gene set.

**Supplementary Figure S3: SETD8 is more expressed in patients at relapse compared to diagnosis.** Boxplot showing SETD8 expression in MM cells of patients at relapse (n=47) compared to diagnosis (n=205).

**Supplementary Figure S4: UNC-0379 inhibits SETD8 and affects MM cells survival and cell cycle**. **(A)** Immunoblot analysis of SETD8, Histone H4 and H4K20me1 protein levels in XG7 and XG25 cells untreated or treated with 3 μM of UNC-0379 for 24 hours. **(B)** Quantitation of cell-cycle distribution of control (untreated) and UNC-0379-treated XG7 and XG25 HMCLs 48 hours after treatment. After short-pulse of BrdU incorporation, cell-cycle was analyzed by FACS using DAPI and anti-BrdU antibody. **(C)** Quantitation of apoptosis in control and UNC-0379-treated XG7 and XG25 HMCLs by flow cytometry with AnnexinV-PE staining and 96h after UNC-0379 treatment. Data shown are mean values ± SD of 4 separate experiments. Statistical analysis was done with a paired t-test. (*) indicates a significant difference compared to control cells using a Wilcoxon test for pairs (P ≤ 0.05).

**Supplementary Figure S5: UNC-0379 treatment did not affect *in vitro* differentiation of human memory B cells*.*** *In vitro* differentiation of memory B cells into plasma cells from 2 different donors. Cells were treated at Day 7 with UNC-0379 at the indicated concentrations and analyzed at Day 10. **(A)** Total cell number was visually quantified. Dead cells were visualized by trypan blue staining and viability was calculated as the ratio of living cells vs total cells. Annexin V+ cells were quantified by FACS. **(B)** Percentage of each population at day 10 (MBC: memory B cells, PrePB: preplasmablasts, PB: plasmablasts, PC: plasma cells). Mock is treated with the same percentage of DMSO as 5 μM sample.

**Supplementary Figure S6: UNC-0379 affects murine MM cells viability.** Measure of viability and apoptosis in primary murine 5T3vv cellular models untreated (cnt) or treated with growing concentrations (from 2.5 to 40 μM) of UNC0379 for 24 hours.

**Supplementary Figure S7: UNC-0379 treatment deregulates gene expression in HMCLs. (A)** Heatmap of RNA-sequencing rlog expression data. Genes deregulated by UNC-0379 and shSETD8 in XG7 and XG25 HMCLs are represented. Expression scale shows low expression levels in blue and high expression levels in red. (**B**) Bar-plot representing the fold expression (UNC-0379 condition over control) of genes related to Fig 1B chart pathways. **(C)** Bar-plot representing the fold expression (UNC-0379 condition over control) of genes related to Fig 1B chart pathways.

**Supplementary Figure S8: SETD8 expression correlation with p53 expression or status.** **(A)** Comparison of *SETD8* expression according to HMCLs *TP53* status. (B) Correlation between *TP53* and *SETD8* expression in HMCLs. *TP53* and *SETD8* expression was obtained from Affymetrix microarrays data previously published.

**Supplementary Figure S9:** **Correlation between *TP53* expression and drug response to UNC-0379 in HMCLs.** IC50 was determined using CTG-based growth assay, *TP53* expression was obtained from Affymetrix microarrays data previously published.

**Supplementary Figure S10:** **UNC-0379 induces genomic instability in XG1 p53-mutant cell line.** **(A)** Immunoblot analysis of indicated proteins in total lysates from UNC-0379-treated (5µM) or untreated XG1 HMCL. β-actin and H2A.X were used as loading controls. **(B)** Cell cycle of control and 48h UNC-0379-treated (5µM) XG1 HMCL was analyzed by flow cytometry using DAPI, BrdU incorporation and labelling with an anti-BrdU antibody. Results are representative of three independent experiments. * indicates a significant difference compared to control cells using a Wilcoxon test for pairs (P ≤ 0.05). (**C**) Apoptosis induction was investigated using AnnexinV-PE staining by flow cytometry.

**Supplementary Figure S11 : UNC-0379 did not alter the steady states levels of nucleolar proteins and the nucleolar localization of fibrillarin. (A)** immunoblot analysis of indicated proteins in shRNA control and p53-depleted XG7 cells treated with the vehicle DMSO or 3 µM of UNC-0379 for 24 hours. Ponceau staining is used as loading control **(B)** immunofluorescence microscopy of fibrillarin localization and DNA (DAPI staining) in XG7 cells depleted or not for p53 at low and higher magnitude as indicated. Scale bar : 10 μm

**Supplementary Figure S12: The lethal synergy between UNC-0379 and melphalan is caused by SETD8 inhibition but does not depend on p53 activation**. (A) immunoblot analysis of SETD8 and tubulin protein levels in total lysates of XG7 cells expressing a doxycycline-inducible shRNA targeting *SETD8* mRNA and treated or not with doxycycline (1µg/ml) for 4 days. **(B)** The graph shows one representative from 3 independent experiments. Cell proliferation analysis was performed by CTG, 4 days after co-treatment with Doxycycline (1ug/ml) to silence SETD8 and various concentrations of melphalan as indicated. **(C)** IC50 values from 3 independent experiments performed as in (B). p-value = 0.0196. (**D**) Measure by flow cytometry of apoptosis induction (AnnexinV-PE staining) in XG7-shControl and XG7-shTP53 HMCLs after 96h of treatment with Melphalan (5μM), UNC-0379-treated (3μM) or the combination of the two drugs.

**Supplementary Figure S13: Immunoblot analysis of histone H2AX and its phosphorylated form γH2A.X (marker of DNA damage) in total lysates from melphalan-resistant XG7 HMCLs.** Cells were treated with melphalan (5μM), UNC-0379-treated (3μM) or the combination of the two drugs for 24h. β-actin was used as loading control.
