# Supplementary material for: Targeting the methyltransferase SETD8 impairs tumor cell survival and overcomes drug resistance independently of p53 status in multiple myeloma
Source: Clin Epigenetics. 2021 Sep 16;13:174. doi: 10.1186/s13148-021-01160-z (PMC8447659; doi:10.1186/s13148-021-01160-z)

Supplementary Figure S1

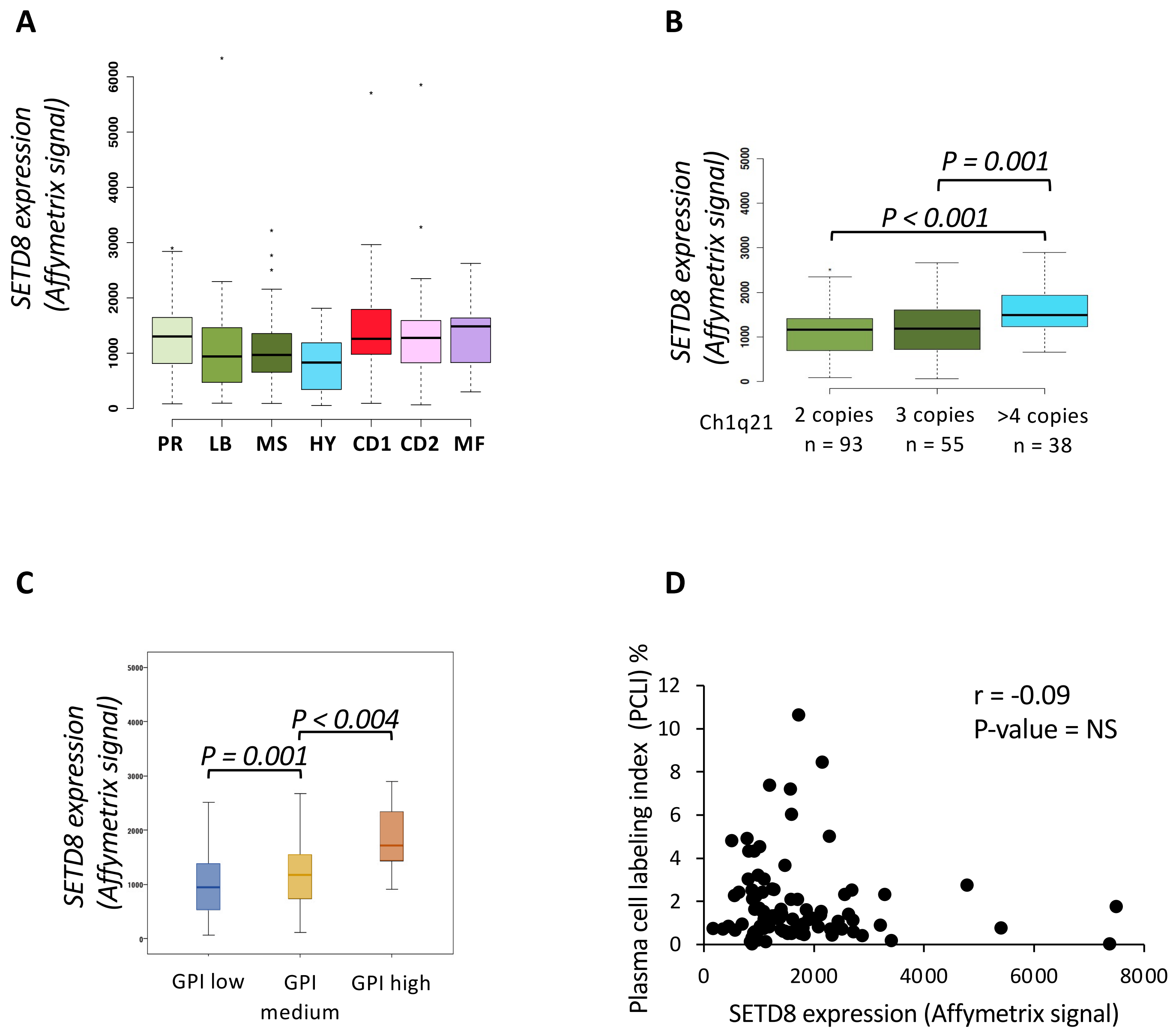

Supplementary Figure S2

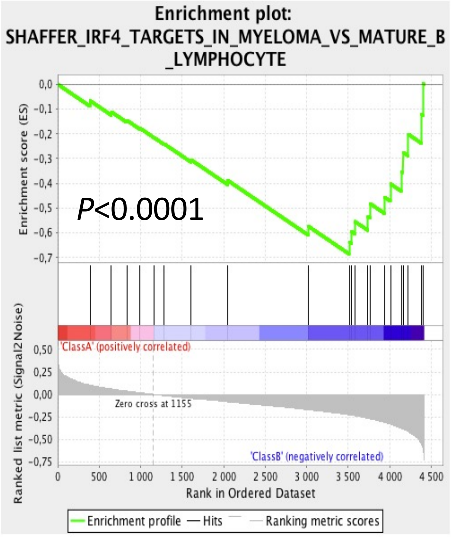

Normalized enrichment score = -2.02

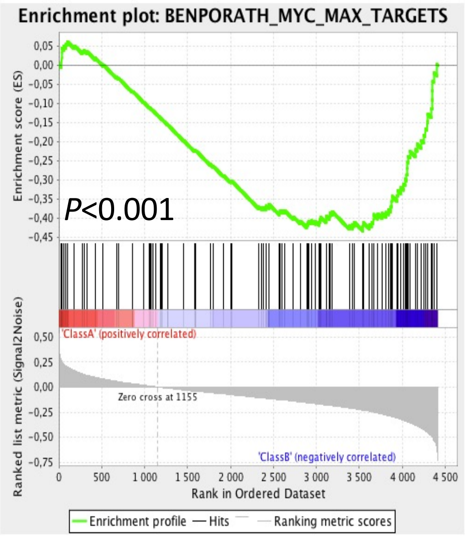

Normalized enrichment score = -1.83

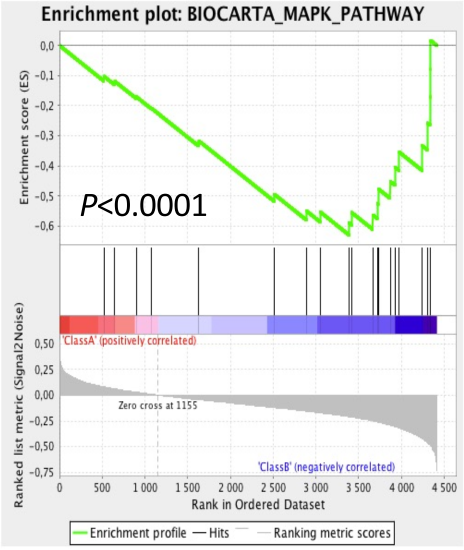

Normalized enrichment score = -1.89

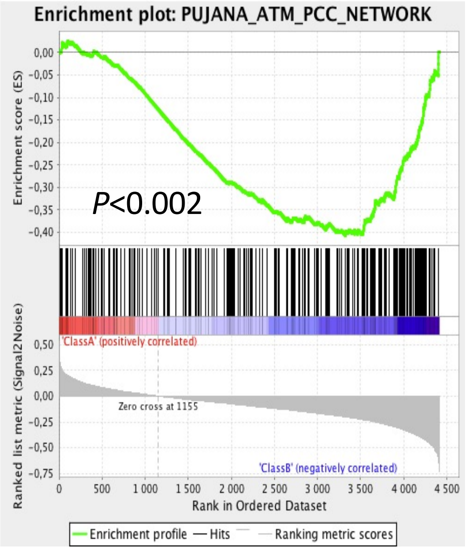

Normalized enrichment score = -1.82

Supplementary Figure S3

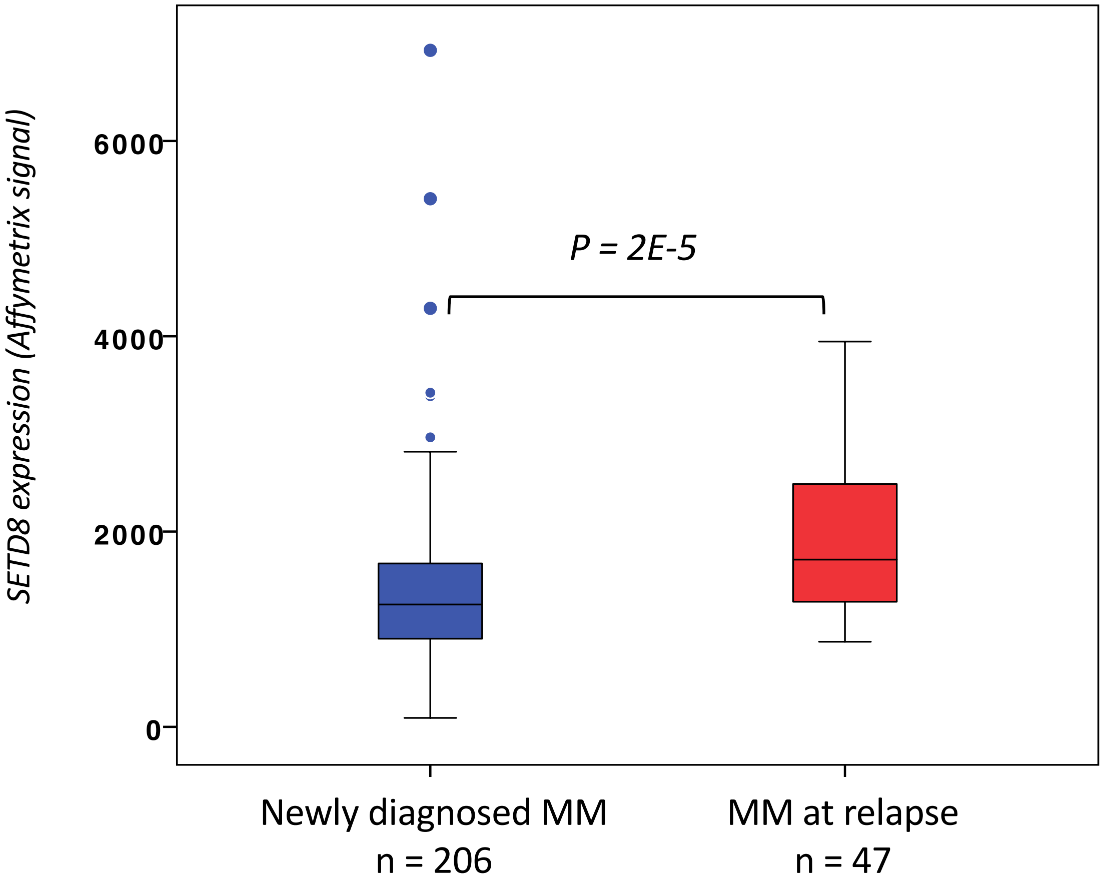

Supplementary Figure S4

A

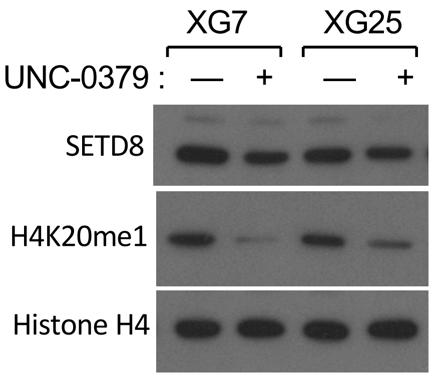

B

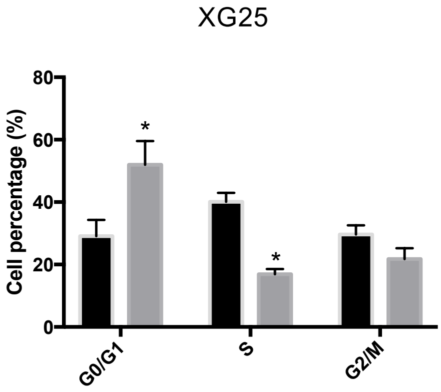

C

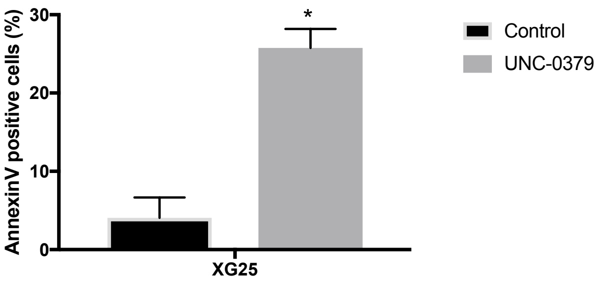

Supplementary Figure S5

A

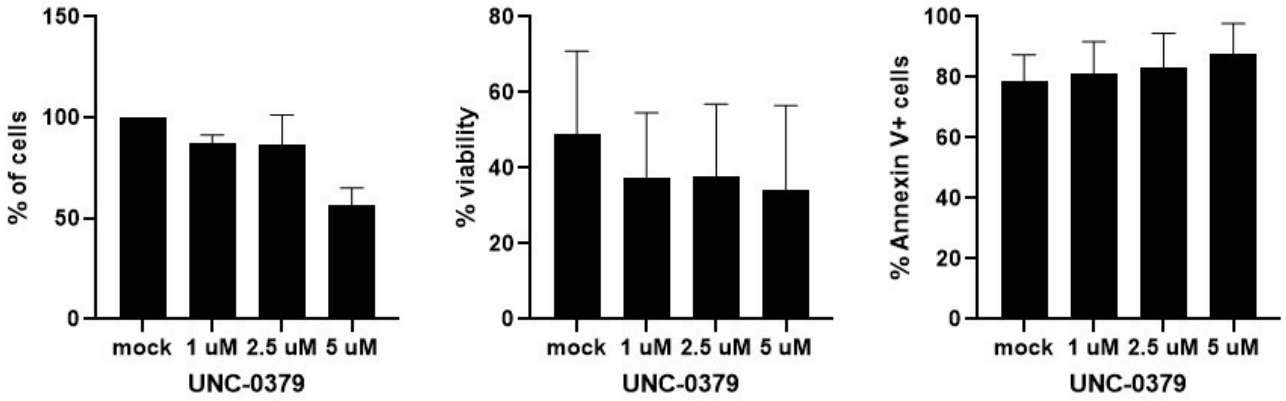

B

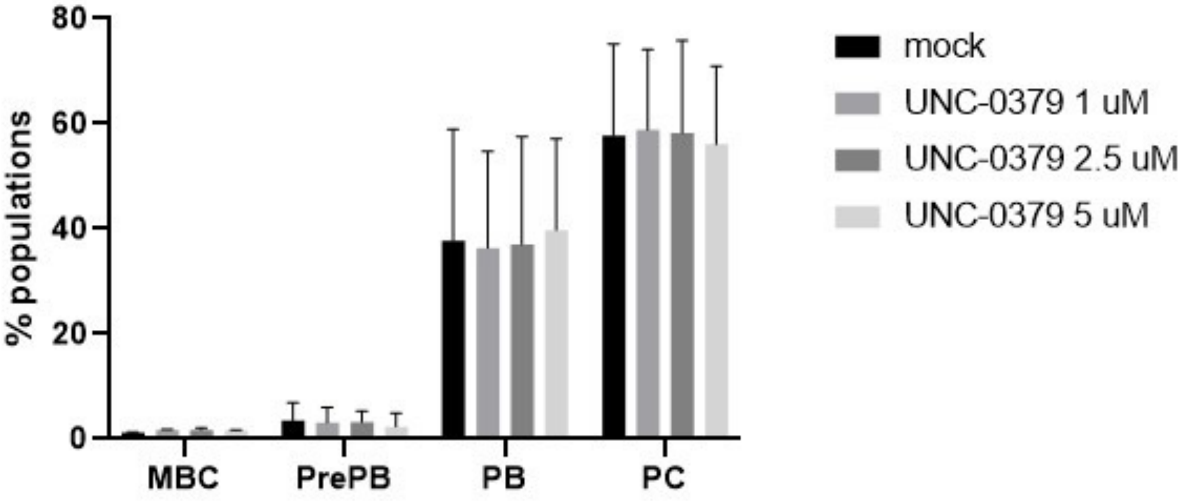

Supplementary Figure S6

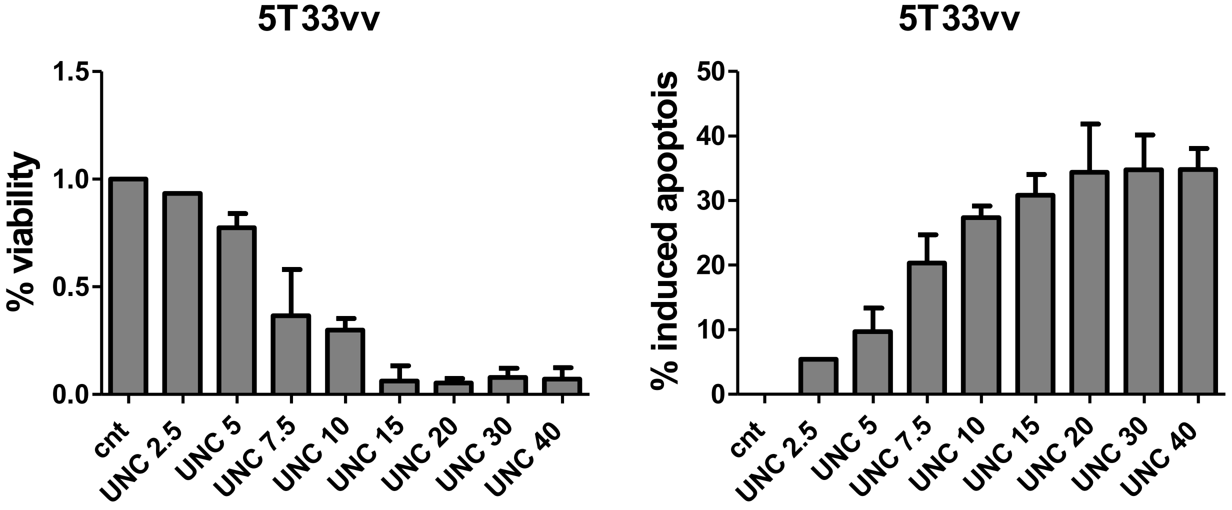

Supplementary Figure S7

A

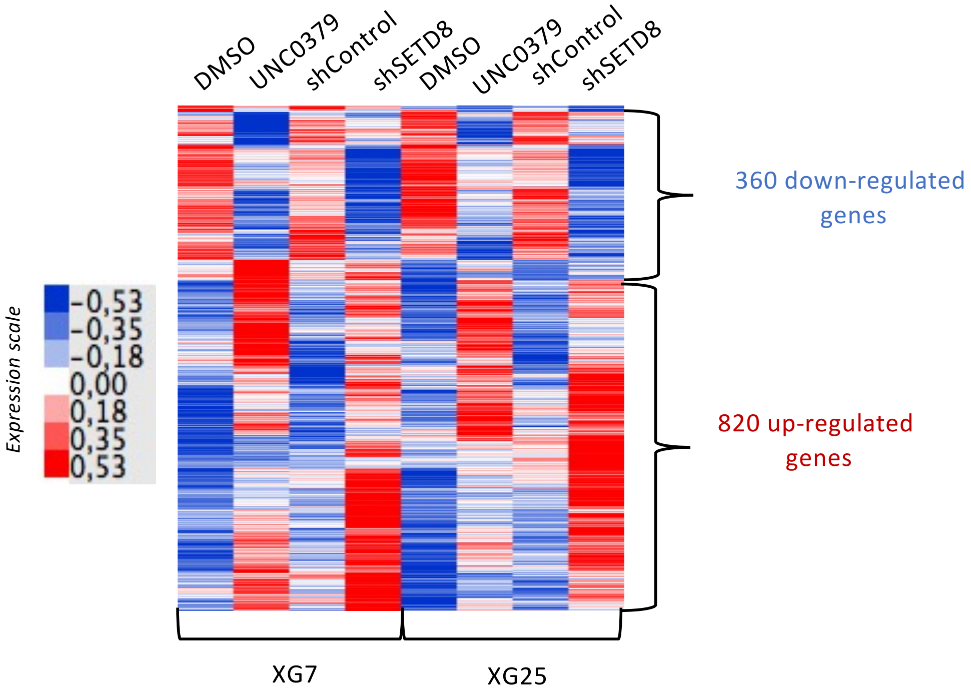

B

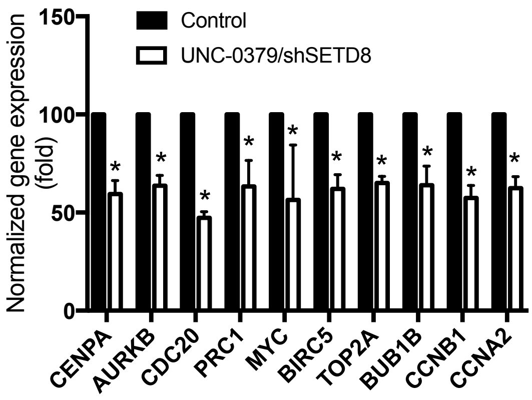

C

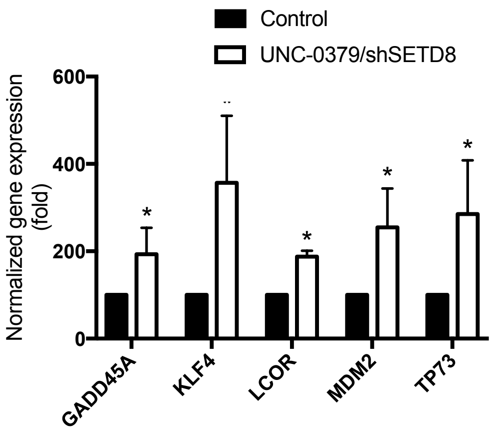

Supplementary Figure S8

A

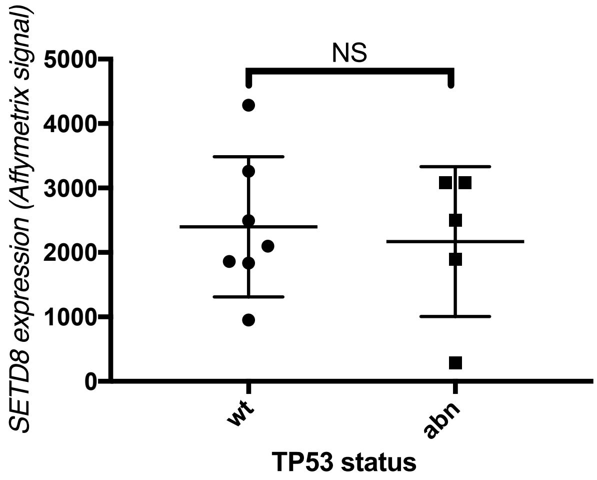

B

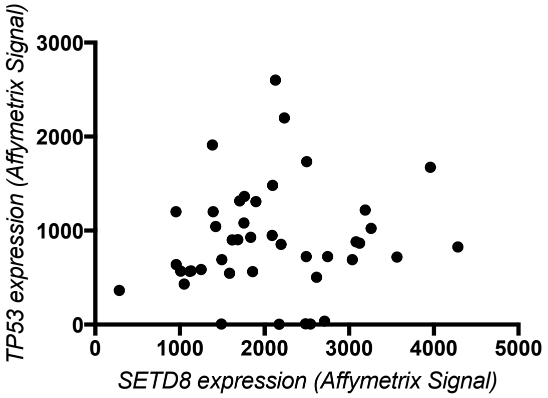

Supplementary Figure S9

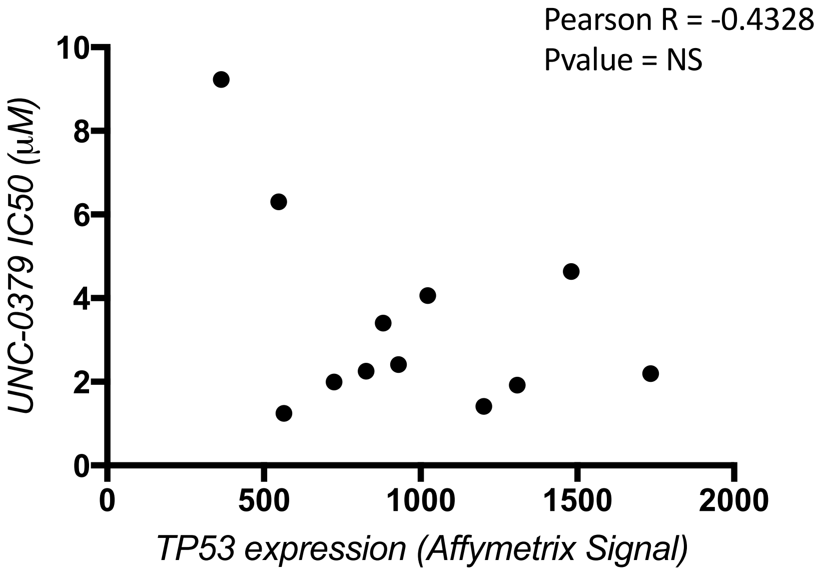

Supplementary Figure S10

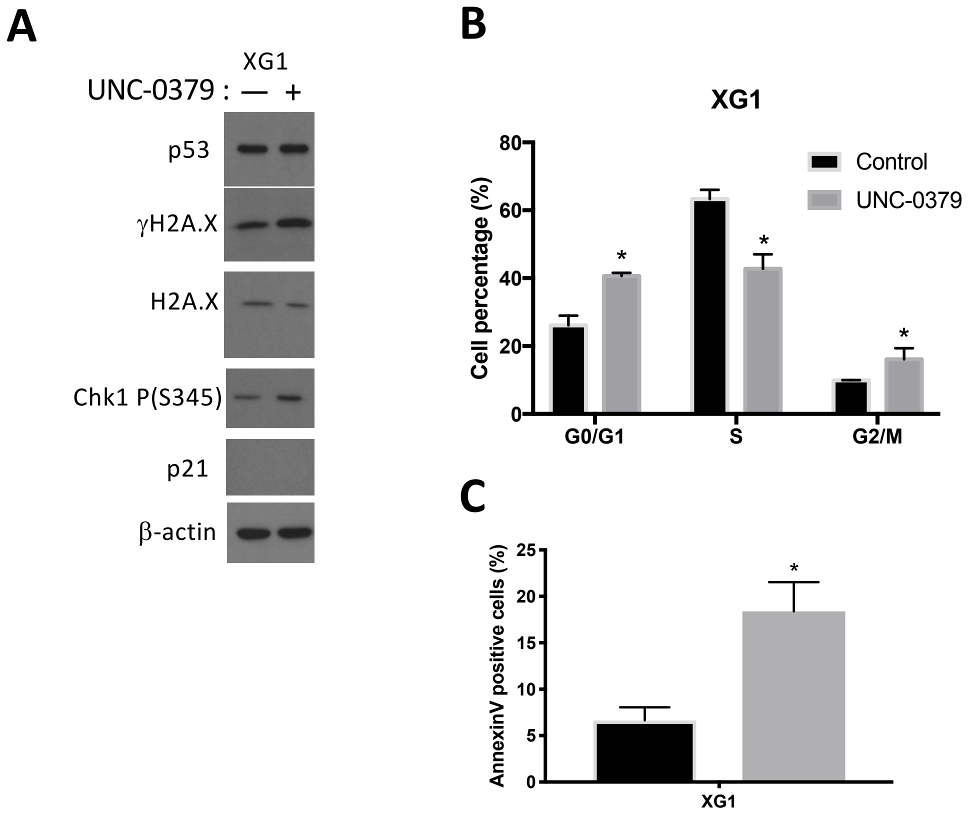

Supplementary Figure S11

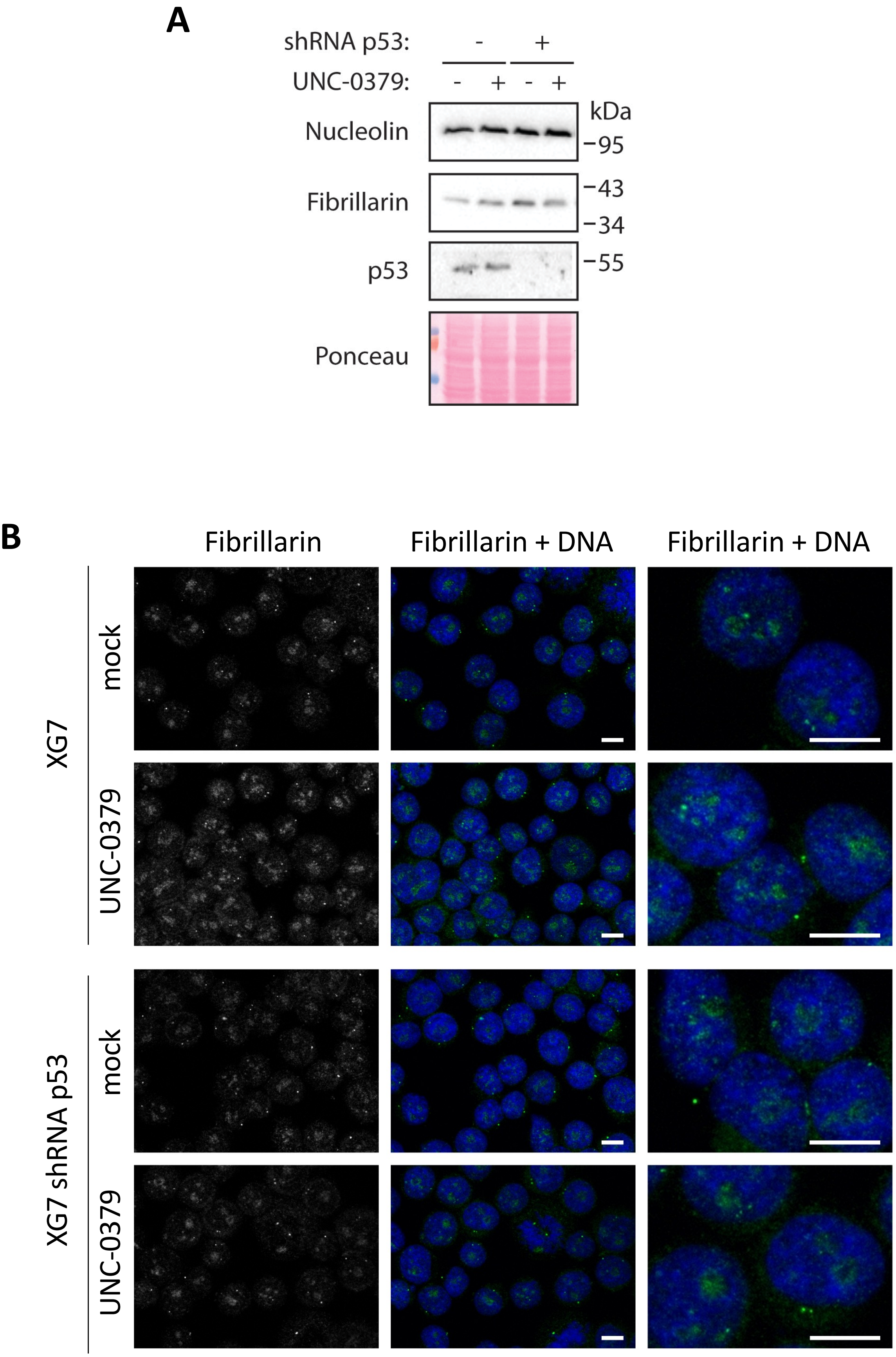

Supplementary Figure S12

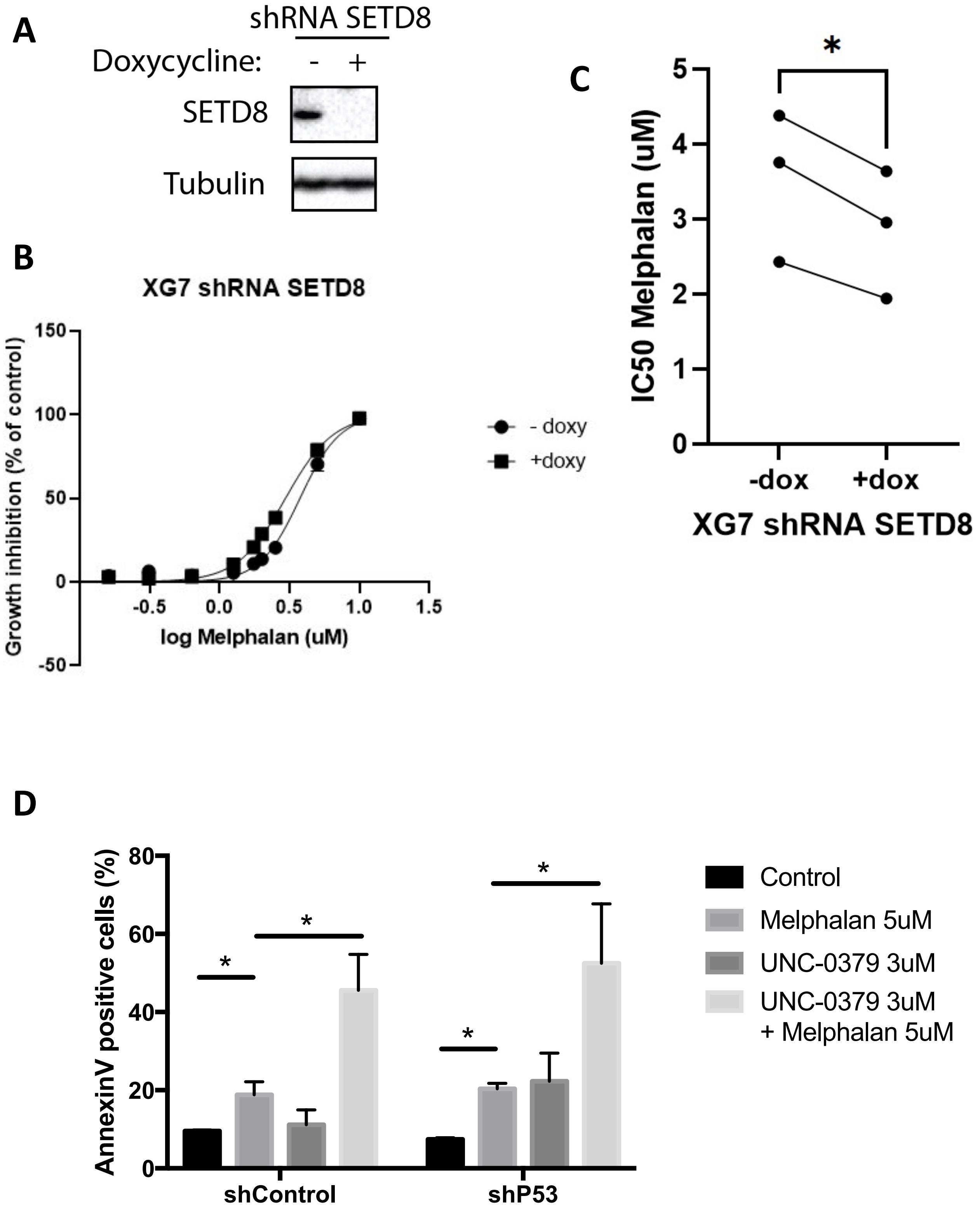

Supplementary Figure S13

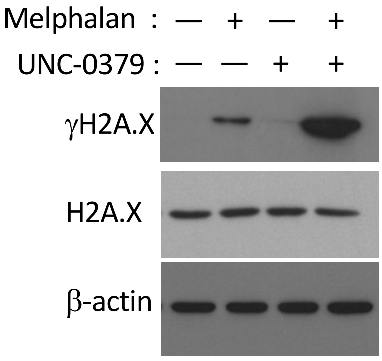

Supplement: Supplementary file 3 — Additional file 3. Supplementary Figure S1: SETD8 expression status in MM patients (A) Gene expression profiling of MMCs of the patients of UAMS-TT2 cohort were used. PR: proliferation, LB: low bone disease, MS: MMSET, HY: hyperdiploid, CD1: Cyclin D1-Cyclin D3, CD2: Cyclin D1-Cyclin D3, MF: MAF, MY: myeloid. (B) SETD8 expression in 186 patients of the UAMS-TT2 cohort showing Ch1q21 copy number aberration. (C) SETD8 expression in MMCs (patients at diagnosis) presenting low, medium or high gene expression-based proliferation index (GPI). (D) Correlation between SETD8 expression and malignant plasma cell labeling index. Plasma cell labeling index was investigated using BrdU incorporation and flow cytometry in 101 patients at diagnosis. Supplementary Figure S2: Gene Signature of MM patients with high SETD8 expression. GSEA enrichment plots with the absolute enrichment p value and the normalized enrichment score of the gene set. Supplementary Figure S3: SETD8 is more expressed in patients at relapse compared to diagnosis. Boxplot showing SETD8 expression in MM cells of patients at relapse (n=47) compared to diagnosis (n=205). Supplementary Figure S4: UNC-0379 inhibits SETD8 and affects MM cells survival and cell cycle. (A) Immunoblot analysis of SETD8, Histone H4 and H4K20me1 protein levels in XG7 and XG25 cells untreated or treated with 3 μM of UNC-0379 for 24 hours. (B) Quantitation of cell-cycle distribution of control (untreated) and UNC-0379-treated XG7 and XG25 HMCLs 48 hours after treatment. After short-pulse of BrdU incorporation, cell-cycle was analyzed by FACS using DAPI and anti-BrdU antibody. (C) Quantitation of apoptosis in control and UNC-0379-treated XG7 and XG25 HMCLs by flow cytometry with AnnexinV-PE staining and 96h after UNC-0379 treatment. Data shown are mean values ± SD of 4 separate experiments. Statistical analysis was done with a paired t-test. (*) indicates a significant difference compared to control cells using a Wilcoxon test for pairs (P ≤ [file 13148_2021_1160_MOESM3_ESM.pdf]
